# Transcriptomic Variations and Network Hubs Controlling Seed Size and Weight During Maize Seed Development

**DOI:** 10.3389/fpls.2022.828923

**Published:** 2022-02-14

**Authors:** Yanzhao Wang, Lihong Nie, Juan Ma, Bo Zhou, Xiaohua Han, Junling Cheng, Xiaomin Lu, Zaifeng Fan, Yuling Li, Yanyong Cao

**Affiliations:** ^1^Henan Provincial Key Laboratory of Maize Biology, Institute of Cereal Crops, Henan Academy of Agricultural Sciences, Zhengzhou, China; ^2^Institute of Industrial Crops, Henan Academy of Agricultural Sciences, Zhengzhou, China; ^3^State Kay Laboratory of Agro-biotechnology and Key Laboratory of Pest Monitoring and Green Management-MOA, China Agricultural University, Beijing, China; ^4^Henan Maize Engineering Technology Joint Center, Henan Agricultural University, Zhengzhou, China

**Keywords:** *Zea mays*, allele-specific expression, hub gene, seed development, seed size, seed weight, *ZmARF12*

## Abstract

To elucidate the mechanisms underlying seed development in maize, comprehensive RNA-seq analyses were conducted on Zhengdan1002 (ZD1002), Zhengdan958 (ZD958), and their parental lines during seven seed developmental stages. We found that gene expression levels were largely nonadditive in hybrids and that cis-only or trans × cis pattern played a large role in hybrid gene regulation during seed developmental stage. Weighted gene co-expression network (WGCNA) analysis showed that 36 modules were highly correlated (*r* = −0.90–0.92, *p* < 0.05) with kernel weight, length, and width during seed development. Forty-five transcription factors and 38 ribosomal protein genes were identified as major hub genes determining seed size/weight. We also described a network hub, *Auxin Response Factor 12* of maize (*ZmARF12*), a member of a family of transcription factor that mediate gene expression in response to auxin, potentially links auxin signal pathways, cell division, and the size of the seeds. The *ZmARF12* mutant exhibited larger seed size and higher grain weight. *ZmARF12* transcription was negatively associated with cell division during seed development, which was confirmed by evaluating the yield of protoplasts that isolated from the kernels of the mutant and other inbred lines. Transient knock-down of *ZmARF12* in maize plants facilitated cell expansion and division, whereas transient silencing of its potential interactor *ZmIAA8* impaired cell division. *ZmIAA8* expression was repressed in the *ZmARF12* over-expressed protoplasts. The mutant phenotype and the genetics studies presented here illustrated evidence that *ZmARF12* is a cell division repressor, and potentially determines the final seed size.

## Introduction

While seeds are well established as a major food source for humans around the world, they are also important raw materials for industry. Seed development, including seed size and weight, is crucial to the life cycle of higher plants, and impacts seed yields. In maize (*Zea mays* L.), seed development contains several phases: early development consisting of double fertilization and syncytium formation, followed by cell differentiation, periods of mitosis and endoreduplication, accumulation of storage compounds, maturation, and desiccation (Wang et al., 2012). It is important to understand the molecular mechanisms regulating the various aspects of seed development in maize, including their size and weight, to improve seed yields.

High-resolution transcriptome technology has been used to monitor variations in gene expression during seed development. Transcriptomes of F_1_ hybrids and their parents allow the investigation of gene expression patterns and variation in regulation, which have been proposed to play a role in heterosis. Two general modes of gene expression in hybrids, i.e., additive and nonadditive, are used to describe gene expression levels in F_1_ hybrids, with respect to the average expression value (mid-parent value) of two parents. Nonadditive patterns in hybrids deviate significantly from the mid-parent values, while additive expression patterns do not. The differences in genetic background, tissues, developmental stages, and methodologies result in nonadditive expression pattern being predominant in many hybrids (Hoecker et al., 2008; Jahnke et al., 2010; Zhao et al., 2019); whereas additive expression is the most common in other known cases (Stupar and Springer, 2006; Paschold et al., 2012; Hu et al., 2016; Zhou et al., 2019).

The relative contribution of cis- and trans-acting regulatory changes can be investigated using allele-specific expression analysis (relative to parental ratios) on F_1_ plants (Zhou et al., 2019). Generally, the predominant roles of cis-effects have often been demonstrated between species, whereas trans-effects are known to play a larger role within species (Wittkopp et al., 2004, 2008; Shi et al., 2012; Wu et al., 2016; Haas et al., 2020; Mattioli et al., 2020). Cis- and trans-effects occur simultaneously, and their compensatory effects facilitate the stability of gene expression an evolutionary timescale (Mattioli et al., 2020). The cis- and trans-acting regulation determine the differences in gene expression. The prevalence of cis-regulatory variation, subject to additive expression, has been observed in multiple tissues in maize (Stupar and Springer, 2006; Hu et al., 2016; Zhou et al., 2019), and in other species (Hughes et al., 2008; McManus et al., 2010; Cubillos et al., 2014; Haas et al., 2020). Nonadditive gene expression occurs when a combination of different alleles contributes to trans-acting interactions in a hybrid. In maize, studies on these hybrids have largely focused on root, meristem, leaf, silk, tassel, and one or two stages of seed development, with respect to seed, embryo, and endosperm tissues. It has not yet been possible to monitor the dynamic changes of additive and nonadditive expression patterns and variations in regulatory throughout the entire seed developmental phase.

Analysis of transcriptome dynamics aids the identification of gene co-expression modules and genes that act as critical network hubs and interpret the biological processes and pathways associated with development. Systems biology approaches, such as co-expression networks have been used to elucidate the patterns of transcriptome organization (Lin et al., 2019; Zhou et al., 2019). Zhang et al. (2016) identified co-expression modules that were highly correlated with seed weight enrichment during ovule development and DNA methylation. Their methodology comprised weighted gene co-expression network analysis (WGCNA) of parental inbred and reciprocal F_1_ hybrids at 14 days after pollination (DAP14) and DAP17. They found that ARF, bZIP, G2-like, MADS-box, and orphan genes were involved in seed size determination. Downs et al. (2013) constructed a developmental gene expression network from microarray transcriptome profiles of 50 maize tissues, including the embryo and endosperm, across different stages of development, and found 24 modules that were strongly associated with tissue types and related biological processes. Sekhon et al. (2014) identified 19 gene co-expression modules for the whole seed, embryo, and endosperm at various developmental stages, and found that one module was rich in zein genes, and that many other genes were significantly and closely related to cell cycle and division, and starch accumulation.

Zhengdan958 (ZD958) is cultivated from Zheng58 (Z58) and Chang7-2 (C72) and has been widely grown in China since the year 2000. Zhengdan1002 (ZD1002) is developed from Zheng588 (Z588) and ZhengH71 (ZH71) in 2015 and improved the yields by 4.4–6.7% over ZD958. Z58 and C72 are representative inbred lines coming from the Reid and SPT heterotic groups, respectively. Z588 and ZH71 are developed from Z58 and C72, respectively. RNA-seq was used to characterize two commercial Chinese varieties of ZD958 and ZD1002, their four parents, and two other F_1_ hybrid lines, at seven seed developmental stages. We analyzed these data to reveal the transcriptome variations between the F_1_ hybrid and parental lines and to identify gene expression patterns and regulatory variations in hybrids during different seed developmental stages. We identified significant co-expression modules, hub genes, and networks that determined maize seed size and weight. We characterized a hub gene, *Auxin Response Factor 12* of maize (*ZmARF12*), which regulates seed size and seed yield in maize. We found that the transcripts of *ZmARF12* were negatively associated with cell division during kernel development; by evaluating protoplasts yields isolated from the kernels of the mutants and the four inbred lines. Transient silencing of *ZmARF12* and its interactor, *ZmIAA8*, in maize plants through virus-induced gene silencing (VIGS) also confirmed their effects on cell division. One of the *ZmARF12* mutants exhibited characteristically larger seed size and higher grain weight. In addition, the transient over-expression of *ZmARF12* in maize protoplast isolated from kernels constrained the expression of *ZmIAA8*. The mutant phenotype and the genetics studies described here demonstrate that *ZmARF12* represses cell division, and potentially plays roles in determining final seed size and yield. This work provides important insights into the gene expression and regulation patterns of commercial hybrids and the molecular networks responsible for maize seed development.

## Materials and Methods

### Plant Materials and Field Trials

The four inbred lines Z58, Z588, C72, and ZH71 and their four F_1_ hybrid combinations ZD958, ZD1002, Z58/ZH71, and Z588/C72, were used in this study. The trials took place in 2017 growing season, on the experimental fields of the Henan Academy of Agricultural Sciences, China. The eight maize lines were arranged in 10-row plots with 15 plants per row, with three replications. The sampling time points for maize seeds were DAP0, DAP8, DAP10, DAP12, DAP20, DAP30, and DAP40. The 100-fresh kernel weight (FKW), 100-dry kernel weight (DKW), ten-kernel length (TKL), and ten-kernel width (TKW) were measured between DAP8 and DAP40 at the aforementioned time points. DKW was determined after drying the samples at 65°C in an oven for 1 week.

Seeds were obtained, with our thanks, from the Maize Genetics Cooperation Stock Center (for *ZmARF12* mutants: UFMu-04333, UFMu-09264, UFMu-09010, UFMu-09505, and UFMu-11213), University of Nebraska, Lincoln [cv. Va35 that were used for Brome mosaic virus (BMV) inoculation].

### RNA Sample Collection and Sequencing

After sampling, the tissues were quickly frozen in liquid nitrogen and stored at −80°C until RNA isolation. Total RNA from whole developing kernels from the four parental lines and four F_1_ hybrids at DAP0, DAP8, DAP10, DAP12, DAP20, DAP30, and DAP40, was extracted using the TRIzol reagent (Invitrogen, Carlsbad, CA, United States) following the manufacturer’s instructions. RNA-seq was constructed using Illumina HiSeqTM4000. After filtering, clean sequence reads were aligned to the B73 reference genome^1^ using HISAT v2.2.1.^2^ Fragments per kilobase of transcript per million mapped read (FPKM) values were estimated using Cufflinks v2.2.1.^3^

Pearson’s correlation (three biological replicates for each genotype) was calculated using the cor.test function from stats in R 4.0.1.^4^ The transformed and normalized gene expression values with log_2_ (FPKM + 1) were used for the principal component analysis (PCA). PCA was performed using the fast.prcomp function from gmodels in R 4.0.1.

### DNA Sample Collection and Resequencing

In order to select high-confidence variants for allele-specific expression analysis using a genomic control, the whole-genome resequencing was applied for four parental lines. During 6–10 leaf stage, five leaves from five individuals of each inbred line were selected, and genomic DNA was extracted using a CTAB method. Whole-genome resequencing was performed on an Illumina Hiseq X10 platform, with a resequencing depth of 30 X. Filtered reads were aligned with the maize B73 reference genome using BWA v0.7.15. Variant calling was performed for multiple-samples using the Genome Analysis Toolkit (v4.0.1.2); those exhibiting segregation distortion or sequencing errors were discarded. The software tool, ANNOVAR (Wang et al., 2010), was used to align and annotate the single-nucleotide polymorphisms (SNPs).

### Identification of Differentially Expressed Genes and Gene Functional Analyses

Differential expression analysis was conducted using the edgeR package (v3.12.1). In each pairwise comparison, the differentially expressed genes (DEGs) were filtered using the expression levels: FPKM > 1, FDR (the Benjamini and Hochberg false discovery rate) < 0.05, |log_2_ fold change| > 1. The DEGs were analyzed using Gene Ontology (GO) with hypergeometric distribution test. The category biological processes of GO terms were retrieved from http://geneontology.org/. Significant biological process terms were defined if the FDR threshold was <0.05.

### Gene Expression Pattern Analysis

Two-tailed homoscedastic variance *t*-tests were used to test if the F_1_ expression were significantly different from the mid-parent expressions. All DEGs with *p* < 0.05 were considered to have been nonadditively expressed; and considered as additive expressions otherwise. The nonadditive DEGs were further categorized into three distinct classes, based on their deviations from the mid-parent value: over-dominance (above the higher inbred parent level), dominance (in-between the inbred parent levels), and underdominance (below the lower inbred parent level).

### Cis- and Trans-Regulatory Expression Assignment

Only SNPs identified by both transcriptome and whole-genome resequencing were used to infer gene regulation patterns. In addition, the genes used to infer the allele ratio of hybrid expression had to meet two criteria: (1) both parents in the three biological replicates had different allele types, and the alleles were heterozygous for each replicate in the hybrids; (2) every SNP was supported by no less than 20 reads in the hybrids and parents. When a gene showed multiple regulatory patterns, one pattern whose proportion was larger than or equal to 60% could be considered the pattern of the gene. The regulatory divergence patterns were assigned using the method described in McManus et al. (2010).

Briefly, the relative allelic expression of each gene was tested in F_1_ hybrid (named H set) and parent (named P set) using chi-square test against the null hypothesis of 1:1 respectively, and compared between F_1_ and parent (named T set) using two-tailed *t*-tests. The difference was defined as significant in any set with the value of *p* < 0.05. The regulatory divergences were further classified into seven patterns using the following criteria:

Cis-only: significant differential expression in P and H set but not in T set.
Trans-only: significant differential expression in P and T set but not in H set.
Cis + trans: significant differential expression in P, H, and T set. The log_2_-transformed allelic expression ratio has the same sign in H and P set.
Cis × trans: significant differential expression in P, H, and T set. The log_2_-transformed allelic expression ratio has the opposite sign in H and P set.
Compensatory: significant differential expression in H and T set but not in P set. The cis- and trans-regulatory divergences compensate each other.
Conserved: no significant differential expression in P, H, or T set.
Ambiguous: all other patterns.

### Identification of Gene Co-expression Modules and Hub-Genes for Seed Size and Weight

Gene co-expression module assignments were determined based on the FPKM data, using the WGCNA protocol (Langfelder and Horvath, 2008). To identify seed size and weight associated modules, we correlated module eigengenes with FKW, DKW, TKL, and TKW from DAP8 to DAP40. For each of the time points, the genes with mean FPKM > 1 for the 24 samples were analyzed. The soft threshold power *ß* was set to nine for all network constructions except for DAP8 (*ß* = 6). The dynamic tree cut algorithm with a minimum module size of 50 genes was used to cut the hierarchal clustering. mergeCutHeight = 0.15 was used to merge similar modules. A significant module was defined if the *p*-value for module-trait associations was <0.05.

The intramodular connectivity of each gene was calculated using function softConnectivity function of the WGCNA package, in R 4.0.1; top 30 genes with high connectivity values (sum of the correlation coefficient of one gene with all other genes within one module) were considered to be hub genes in one module. The gene networks of the hub genes were visualized using Cytoscape v3.6.1.

### Plant Cultivation and Auxin Treatment

*Nicotina. benthamiana* Domin. plants were grown in growth chambers at 24/22°C (day/night) with a 16 light, 8 h dark cycle as described previously (Chen et al., 2017).

Every sample of five maize (*Z. mays L*. inbred lines B73 or Va35) seeds was placed between two sheets of germination paper, as previously described in Ortiz-Ramírez et al. (2018). Each germination paper cylinder was then transferred into a glass tube (150 × 50 mm in diameter) filled with an 800 × dilution of Peters Pro 20–20–20 water-soluble fertilizer (ICL specialty fertilizers, Summerville, SC, United States) to reach 1/4 of the paper height, and incubated in a growth chamber at 27/24°C (day/night) with a 16/8 h light/dark cycle, and approximately 50% humidity.

The auxin treatment was conducted by transferring the roots of the plants into the Hoagland’s solution containing 5 μM NAA (Sigma, Saint Louis MO, United States) solution. The primary roots of αNAA exposure and the control plants were harvested at 0, 1, 2, and 3 h post-treatment, respectively.

### Real-Time RT-PCR

Total RNA was purified from different samples using TRIzol reagent (Invitrogen, Carlsbad CA, United States), and then treated with RNase-free DNase I (TaKaRa, Dalian, China). The first-strand cDNA was synthesized using 2.0 μg of total RNA per 20 μl reaction and an oligo (dT) primer. Ten-fold diluted cDNA, a set of gene-specific primers (Supplementary Table 2) and a FastSYBR mixture (CWBIO, Beijing, China) were mixed for qPCR to determine the accumulation levels of the maize genes on an ABI 7500 Real-Time PCR system (Applied Biosystems Inc., Foster City, CA, United States). The expression level of *ZmUbi* mRNA was determined and used as an internal control. The relative expression level of each gene was calculated using the 2^–ΔΔCt^ method (Livak and Schmittgen, 2001). Differences between the treatments were then analyzed using Student’s *t*-tests. All experiments were carried out at least three times.

### Transient Silencing of *ZmARF12* and *ZmIAA8* Through Virus-Induced Gene Silencing

The BMV-derived VIGS system was previously described (Cao et al., 2012; Zhu et al., 2014). DNA fragments of 222, and 186 bp, representing the *ZmARF12* and *ZmIAA8* genes, were amplified using specific primer pairs (Supplementary Table 2). The resulting constructs of BMV-*ZmARF12*, BMV-ZmIAA8, and the control BMV-GFP, were then transformed into the *Agrobacterium tumefaciens* strain C58C1. The *N. benthamiana* leaves were infiltrated with *A. tumefaciens* cultures and collected for BMV virion preparation, as described previously (Zhu et al., 2014).

The third leaves of the three-leaf-stage (2 weeks old) Va35 plants were rub-inoculated with approximately 20 μg of partially purified BMV virion. More than 20 seedlings were used for each treatment, and the inoculated plants were grown inside a growth chamber at 18/20°C (day/night) for 7–14 days before being challenged with auxin treatments, or harvested for protoplasts isolation (Zhu et al., 2014). Systemic infection leaves (or equivalent leaves from mock-inoculated plants) from BMV-*ZmARF12*, BMV-ZmIAA8, or BMV-GFP inoculated plants were harvested from individual plants at 7- and 14-d post-inoculation (dpi), and subjected to qRT-PCR to evaluate the efficiency of the gene silencing.

### Quantification of Cell Area and Number of Cell Layers

The maize leaves or kernel pellicles were collected to quantify the cells per square and the number of cell layers, according to a previously described method (de Jong et al., 2015). Fresh leaf disks or pellicle tissues, which were 7–8 mm in diameter, were fixed in formalin-acetic acid-alcohol solution (5% formaldehyde solution, 5% acetic acid, and 45% anhydrous ethanol), and dehydrated with an ethanol series prior to being embedded in Technovit. Sections of 4 μm thick leaf disks or kernel pellicles were then stained with a toluidine blue solution, respectively. The sections were observed and scanned with 3D HISTECH Pannoramic MIDI II scanner (Budapest, Hungary), and micrographs were made with CaseViewer™ digital microscopy system (3D HISTECH). These micrographs were employed for further analysis with the CaseViewer 2.4 application.

For analysis of the Va35 leaf disk, randomly selected 20× micrographs were used to estimate the number of cell layers, by drawing two lines across the central vascular bundle (L1) and the common vascular system (L2), respectively. The number of cells along the two lines were scored. Whereas for the analysis of the cell numbers and number of cell layers within the pellicle, two lines were drawn across the outermost layers of the epidermis and along the longitudinal direction of the pellicle, separately. The total numbers of cells along the lines were numbered.

### Maize Protoplasts Isolation and Transfection

The maize kernels were aseptically dissected from greenhouse-grown ears harvested DAP10 and subjected to protoplasts isolation and transfection using the previously described protocols (Ortiz-Ramírez et al., 2018; Hu et al., 2020). The prepared protoplasts were transfected with pGFP-ARF12 plasmid and incubated at 25°C for 12 h, then the protoplasts were harvested by centrifuging at 500 × *g*, and used for real-time RT-PCR to evaluate the expression profiles of *ZmARF12* and its interactor *ZmIAA8* as previously described (Cao et al., 2012).

## Results

### Morphological Dynamics at Different Seed Developmental Stages

**Figure 1:**
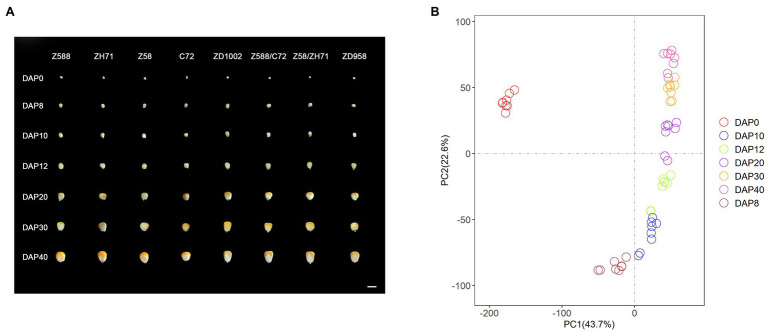
**(A)** The geometry size, shape characteristics of the four inbred lines and the four hybrids were photographed at different days post pollination. Z588, Zheng588; ZH71, ZhengH71; Z58, Zheng58; C72, Chang7-2; ZD1002, Zhengdan1002; and ZD958, Zhengdan958. DAP0–DAP40 representing different days (0, 8, 10, 12, 20, 30, and 40 days) after pollination, respectively. Scale bar = 1 cm. **(B)** Principal component analysis (PCA) of the RNA-seq data at seven developmental stages. Mean fragments per kilobase of transcript per million mapped read (FPKM) values of three biological replicates are used for each genotype at each developmental stage. DAP represents days after pollination.

Seed morphology showed that ZD1002 and its paternal parent ZH71 exhibited larger seed size and heavier weights than ZD958 and C72, respectively, during the seven seed developmental stages (Figure 1A). ANOVA and Duncan’s multiple comparisons of FKW, DKW, TKL, and TKW for DAP8–40 stages further revealed the differences between the seed morphological characteristics of ZH71 and C72 were obvious during the investigation, whereas the differences between Z588 and Z58 were comparatively smaller (Supplementary Figure 1; Supplementary Table 1). ZD1002 and ZD958 showed consistently significant differences in terms of FKW and TKL during seed developmental stages. Four F_1_ hybrids showed significantly increased seed size and weight compared with their respective inbred parents.

### Overview of Transcriptome Analysis of Eight Genotypes

To understand dynamics of the gene expression during seed developmental stages, 168 RNA libraries from eight lines with three replicates at seven stages were constructed. A total of 27,377 maize gene models were expressed with FPKM value ≥ 1 in at least one sample. The number of expressed genes ranged from 18,442 to 21,276 for 168 samples. The correlation of three biological replicates were high for eight maize lines at each developmental stage (0.9575–0.9999; Supplementary Figure 2).

The PCA revealed that the 56 samples (eight genotypes at seven stages) were assigned to seven stages, and that DAP0 was clearly distinguished from other six stages (Figure 1B). DAP8 was also found to be distinguishable from the other six stages. Except for DAP0 and DAP8, the neighboring two DAP stages showed similarities. Moreover, two stages were often overlapping, such as DAP10 and DAP12, and DAP30 and DAP40 (Figure 1B). The pairwise comparisons of the two neighboring stages also identified major transcriptional differences between DAP0 and DAP8, and DAP8 and DAP10 for eight genotypes (Supplementary Figure 3A).

In line with phenotypic variations, the number of DEGs between Z588 and Z58 (72–199) was much lower than that between ZH71 and C72 (408–810) across all developmental stages (Supplementary Figures 3B,C). DNA resequencing revealed that ~7% SNPs are different between Z588 and Z58, whereas the proportion of differences between ZH71 and C72 was ~14% (Supplementary Figure 4). Differential expression analyses showed that the transcriptome profiles of ZD1002 and ZD958 were similar to that of their maternal parents during their early developmental stages (DAP8–12) but closer to paternal parents at the later stages (DAP20–40; Supplementary Figure 3D). In general, the total number of DEGs (4700–5,379) between F_1_ hybrids and paternal parents was slightly higher than that between F_1_ hybrids and maternal parents (4677–5,026; Supplementary Figure 3D). These indicated that paternal parents might have a relative higher contribution to the differences between F_1_ hybrids.

The number of DEGs between two commercial varieties ZD958 and ZD1002 varied from 162 to 1,050 during the seven stages (Supplementary Figure 3E). Compared with ZD958, more downregulated genes were observed in ZD1002 across all developmental stages (Supplementary Figure 3E). The two hybrids showed the largest differences at DAP0, followed by DAP12 and DAP40. The former DEGs were most significantly involved in four terms related to photosynthesis associated processes (Supplementary Figure 5). At DAP12 and DAP40, DEGs between ZD1002 and ZD958 were significantly involved response to heat and temperature stimuli (Supplementary Figure 5).

### Gene Expression Levels Are Largely Nonadditive in Hybrids

The significantly increased differences with respect to seed size and weight in F_1_ hybrids relative to parental lines showed seed heterosis. The differences between F_1_ expression and mid-parent expression levels allow to the assessment of additive and nonadditive patterns. For ZD1002, a large proportion of DEGs (55.38–78.15%) exhibited nonadditive expression at all seven stages (Supplementary Figure 6A). For ZD958 and the two other hybrid crosses, most of DEGs (63.41–85.27%) displayed additive expression at DAP0, whereas differentially expressed profiles were more likely to exhibit nonadditive patterns (56.65–80.23%) for the remaining stages (Supplementary Figures 6B–D). Among nonadditive DEGs, a relative high portion of dominance mode were observed in most stages of the four F_1_ hybrids. For ZD1002, 53.36% of DEGs exhibited over-dominance pattern at DAP12, which was much higher than that of dominance (4.60%) and under-dominance (7.11%). The joint expression patterns of over-dominance and dominance or under-dominance and dominance were also found in our investigation. These supported those multiple modes of gene action with heterosis during seed development. In addition, the transitional changes and proportions of gene expression patterns might result in the phenotypic differences between ZD958 and ZD1002 across seed development.

### Regulatory Divergences During Seed Development

**Figure 2:**
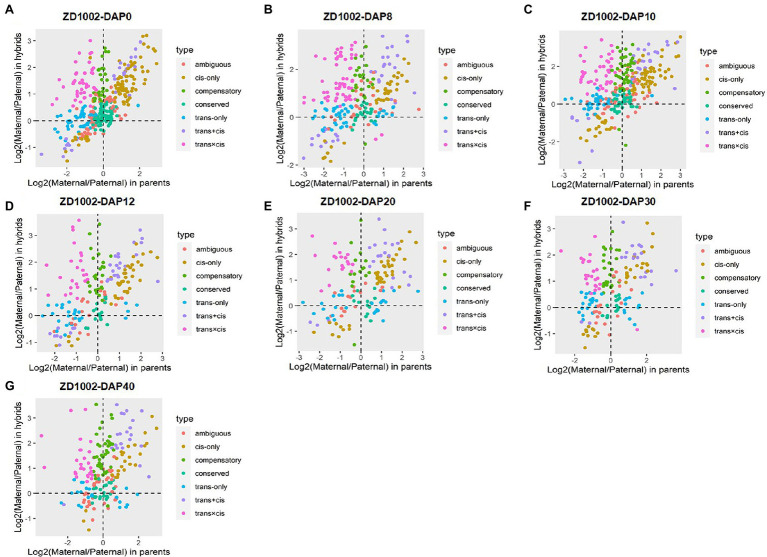
Plots of allele-specific expression levels in Zhengdan1002 (ZD1002) and its parental data set at seven developmental stages. **(A–G)** represent 0 days after pollination (DAP0), DAP8, DAP10, DAP12, DAP20, DAP30, and DAP40, respectively. Each point represents a gene and is color-coded according to the types of regulatory divergence.

**Figure 3:**
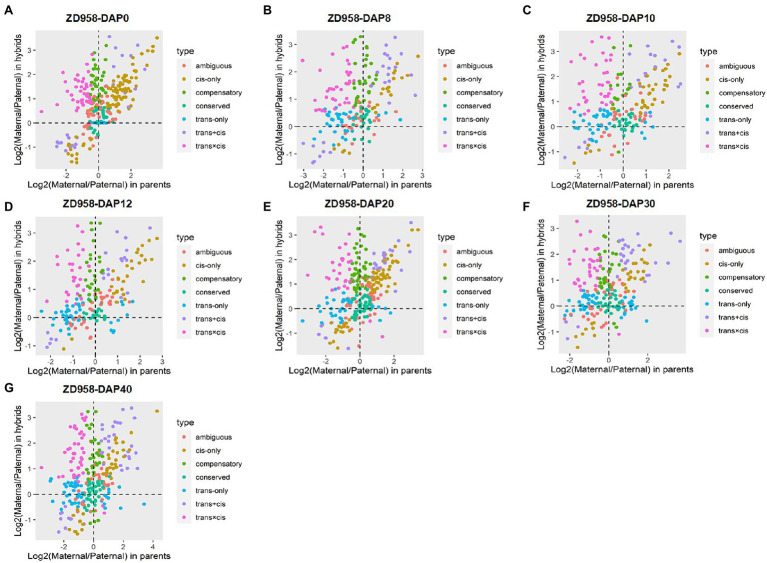
Plots of allele-specific expression levels in Zhengdan958 (ZD958) and its parental data set at seven developmental stages. **(A–G)** represent DAP0, DAP8, DAP10, DAP12, DAP20, DAP30, and DAP40, respectively. Each point represents a gene and is color-coded according to the types of regulatory divergence.

The sources of regulatory variation of genes can be inferred based on their allelic expression ratios in the F_1_ hybrids and parents. In this context, DEGs between parents and hybrids whose SNPs were co-detected in genomic and transcriptomic levels were used to infer regulatory divergence assignment. Here, we showed the regulatory divergences between the two commercial varieties and further revealed their regulatory differences during seed development. For ZD1002, the cis-only regulatory difference was the main type at DAP0, DAP10, DAP12, and DAP20, accounting for 21.55–26.85% of DEGs (Figures 2A,C–E; Supplementary Figure 7A). For ZD958, the cis-only divergence type also accounted for the highest proportion (24.50–30.96%) of DEGs at DAP0 and DAP20 (Figures 3A,E; Supplementary Figure 7B). Three patterns including trans-only, trans + cis, and trans × cis contributed to the gene expression at DAP8 in ZD1002 (Figure 2B). Trans-only and compensatory were the main regulatory patterns at DAP8, DAP30, and DAP40 in ZD1002, respectively, whereas the proportion of trans × cis pattern was the highest at DAP12, DAP30, and DAP40 in ZD958 (Figures 2, 3; Supplementary Figure 7).

### Co-expression Modules Highly Correlated With Seed Size and Weight

Seed size and weight are important traits of seed yield, and are direct reflections of seed development. To identify specific genes that are highly associated with seed size and weight, we constructed co-expression networks for each stage and correlated co-expression modules with seed size and weight related traits. At DAP8 and DAP10 stages, four modules were found to only be significantly correlated with TKW (*r* = −0.49–0.48, *p* < 0.05), and were considered a TKW-specific module (Supplementary Figures 8A,B). From the DAP12 to DAP40, 31 modules significantly correlated with seed size and weight were identified (Supplementary Figures 8C–F). The number of modules ranged from seven to nine for each stage. The correlation coefficient of these module-traits was high (−0.90 to 0.92). Ten of the 31 modules were significantly correlated with all four traits, with correlation coefficients between −0.88 and 0.92.

### Hub Genes of Seed Size and Weight and Their Potential Networks

**Figure 4:**
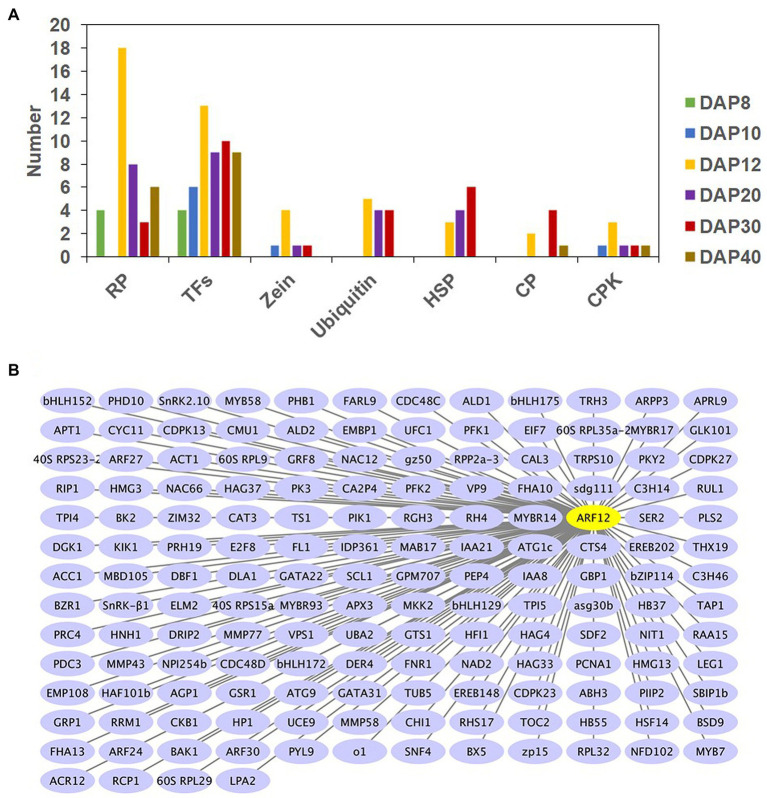
Number of hub genes associated with seed size and weight at six developmental stages **(A)** and gene networks of ARF12 **(B)**. RP, TF, ubiquitin, HSP, CP, and CPK are abbreviations of ribosomal proteins, transcription factors, ubiquitin associated proteins, heat shock proteins, chaperone proteins, and calcium-dependent protein kinase, respectively. DAP8–40 represents 8–40 d after pollination, respectively. Cytoscape representation of co-expressed genes with edge weight ≥ 0.20 is shown. Only genes with annotations are displayed.

Hub genes are those that show the most connections in a network as indicated by their high correlation values, and they may play important roles in the determination of seed size and weight. Forty-five TF-encoding genes were identified as network hubs from DAP8 to DAP40 (Figure 4A). For example, there were five genes from ARF families (ARF3, ARF12, ARF14, ARF18, and ARF28), four MYBs (MYB44, MYB56, MYB73, and MYB79), three MYBRs (MYBR63, MYBR69, and MYBR70), and two bZIPs [abscisic acid-insensitive 5 (ABI5) and opaque2 heterodimerizing protein2 (OHP2); Supplementary Figure 9]. Twenty-five pairs of hub TFs showed potentially interactions with each other (the weight value of 0.11–0.29; Supplementary Figure 9).

Except for DAP10, 38 hub genes encoding ribosomal protein were found from DAP8 to DAP40 (Figure 4A; Supplementary Figure 10). There were 18 RP genes at DAP12, and among them 16 RP genes were found in the sienna3 module. Based on their gene networks, most of the hub RP genes were found to have high connections with each other, as 118 pairs of hub RP genes had high interactions, and their weight values ranged from 0.11 to 0.36 (Supplementary Figure 10). In the sienna3 module of DAP12, 103 pairs of hub genes encoding for RP were connected (0.11–0.20). Apart from inter-connections between hub RP genes, another 66 RP genes also showed interactions with these hub genes (Supplementary Figure 10). In addition, 49 TF genes including 11 hub TFs interacted with the 38 hub RP genes (Supplementary Figure 10).

Some zein genes, E3 ubiquitin-protein ligase, ubiquitin conjugating enzyme, chaperone proteins, and calcium-dependent protein kinases were also considered to be hub genes for seed size and weight (Figure 4A). Their inter-connections were also observed in our investigation (Supplementary Figures 11–14). The identification of hub genes and their networks could help to identify potential cross-talk in the regulation of maize seed development.

### Transcription Profile of Hub Gene *ZmARF12* and Its Potential Interactor *ZmIAA8*

Auxin regulates cell division and is further involved in almost all the processes of plant growth and development, including seed size determination (Sun et al., 2017). The interactions between ARFs and IAAs is the key particular of auxin regulation (Korasick et al., 2014).

In *Arabidopsis*, ARF2 was thought to be negatively regulates auxin signal pathway and seed size through downregulation the cell division in the integument region of endosperm (Schruff et al., 2006). BIG GRAIN1, a positive regulator of auxin response in rice that involved in auxin transport, leads to significant increasement of seed size and plant biomass, seed weight, and finally the yield (Liu et al., 2015). However, the mechanisms underlying ARF and IAA control of seed development remains elusive.

Through WGCNA, we found that ARF12 was a network hub for seed size and weight (Figure 4B; Supplementary Figure 9); we have hence demonstrated the roles of ARF12 in determining maize seed size and weight. The mRNA accumulation of the *ZmARF12* in different tissues was observed, and real-time RT-PCR showed that it was relatively higher in the ear and tassel than in other tissues (Supplementary Figure 15A). To determine the response of *ZmARF12* to the exogenous auxin treatments, the expression levels in the seedling roots at different time points were evaluated after being subjected to 5 μM NAA solution using real-time RT-PCR. The results indicated that *ZmARF12* was upregulated in the first 2 h of treatments, but downregulated after 3 h of auxin treatment. Intriguingly, the mRNA abundance displayed a similar variation tendency in the mock treatment (Supplementary Figure 15B).

To explore the potential roles of *ZmARF12* in regulating the processes of grain production, we analyzed its expression levels at different seed developmental stages. The quantitative RT-PCR data showed that the transcriptional level of *ZmARF12* was rapidly reduced in the first 20 days, then reached a relatively stable level in the four hybrid lines and paternal lines C72 and ZH71. In contrast, the *ZmARF12* transcript level in the maternal lines Z58 and Z588 were consecutively upregulated with the embryo development, except for a slight decrease at DAP30 (Supplementary Figure 15C). The protein–protein interaction networks that possessed ARF12 had significant interactions with Aux/IAA-transcription factor 8 (IAA8), IAA26, and IAA28 (Supplementary Figure 16). IAA8 also showed connections with ARF12 in the WGCNA, with a weight value of 0.23 (Figure 4B). The expressions of *ZmIAA8*, in the four inbred lines and the four hybrids exhibited similar patterns, and were upregulated over the first 8 days, and then downregulated across the early stages (DAP8 to DAP30) of ear fertilization (Supplementary Figure 15D).

### Transient Silencing of *ZmARF12* Facilitates Maize Cell Division Activity

**Figure 5:**
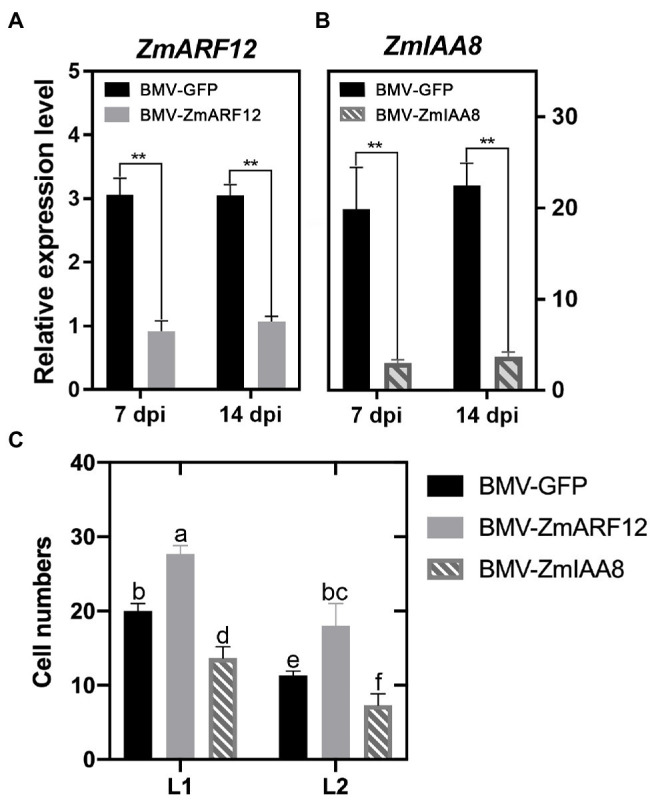
Transient silencing of *Auxin Response Factor 12* of maize (*ZmARF12*) facilitates the cell division of maize leaves. The 7-day-old cv. Va35 maize plants were inoculated virions of either the chimeric brome mosaic virus (BMV-GFP) or BMV-GFP with a *ZmARF12* insert (BMV-ZmARF12) or a *ZmIAA8* insert (BMV-ZmIAA8) to knock-down *ZmARF12*, *ZmIAA8* using brome mosaic virus (BMV) induced gene silencing, respectively. **(A,B)** The first and second systemic leaves above the virus-inoculated leaves were harvested for analysis of the silencing efficiency of *ZmARF12*, *ZmIAA8* at 7 days post-inoculation (dpi) and 14 dpi, respectively. Each value is shown as the average of six independent experiments ± SD. ^**^p < 0.01 (according to a paired Student’s *t*-test); ns, not significant. **(C)** Quantification of cell number per surface unit and number of cell layers of the second systemic leaves above inoculated leaves of *ZmARF12*-, *ZmIAA8*-silenced and control plants. Values are the means ± SD, different letters indicate significant difference (*p* < 0.05) as determined by Tukey–Kramer test.

To understand the potential roles of *ZmARF12* in regulating kernel development. The cell size was evaluated by examining the number of cells per surface unit and the number of cell layers in the leaves of *ZmARF12* in transient silenced plants. We used a BMV-based VIGS system to knock down *ZmARF12* expression. The *ZmARF12* VIGS vector BMV-*ZmARF12* and control plasmid BMV-GFP were introduced into *N. benthamiana* to replicate the BMV virion. The Va35 plants were inoculated with the purified virus particles to transiently silence *ZmARF12* as previously reported (Cao et al., 2012; Zhu et al., 2014). The qRT-PCR results indicated that the expression of *ZmARF12* in the *BMV-ZmARF12* inoculated plants was about 30–35% that of the control plants (Figure 5A). In the transient silenced plants, the expression levels of *ZmARF11*, *ZmARF23*, and *ZmARF24*, which share 42.30, 49.96, and 61.17% homology with *ZmARF12* (Xing et al., 2011; Supplementary Figure 17), were not affected (Supplementary Figure 18). These results demonstrated that silencing had been successfully achieved. Additionally, the transient silencing of *ZmIAA8* facilitated the expression of its potential interacting gene, *ZmARF12* (Figure 5A; Supplementary Figure 18).

At 14 dpi, the second systemic leaves above the inoculated leaves were harvested for analysis of cell expansion and division. The *ZmARF12*-silenced plants had more cells, and more cell layers, compared to those in the control plants (Figure 5C; Supplementary Figure 19). In contrast, transient silencing of *ZmIAA8* impaired the cell division in the systemic leaves (Supplementary Figure 19). These findings suggested that *ZmARF12* might repress cell division during maize leaf growth.

### *ZmARF12* Might Be a Regulator of Cell Division During Seed Development

**Figure 6:**
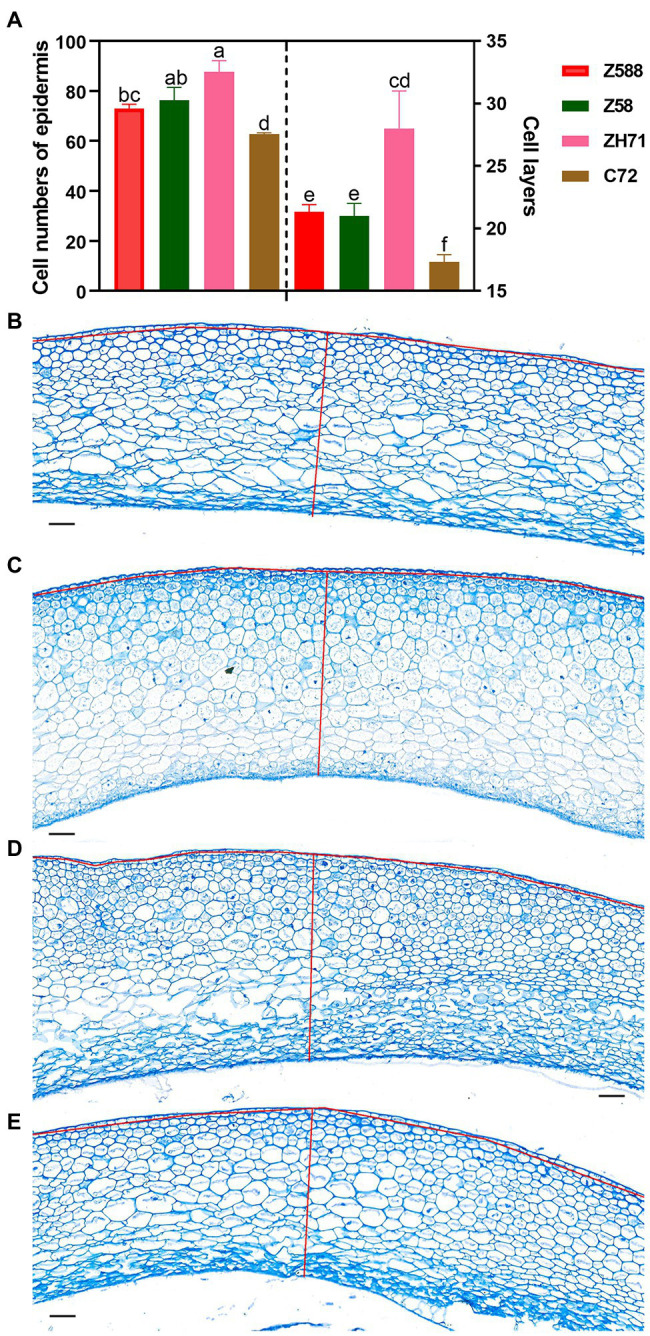
Cell division of the kernel pellicle of the four inbred lines at early seed developmental stage. **(A)** To determine the cell number and number of cell layers in the kernel pellicle, kernels were collected from plants that grown in three different blocks with three replicates per genotype (Z588, Zheng588; ZH71, ZhengH71; Z58, Zheng58; and C72, Chang7-2) per block at 10 d after pollination. Each value is shown as the average of six independent experiments ± SD. Different letters indicate significant difference (*p* < 0.05) as determined by Tukey–Kramer test. Micrographs of the kernel pellicle from Z588 **(B)**, Z58 **(C)**, ZH71 **(D)**, and C72 **(E)**. For estimation of the numbers of cells and layers, the number of cells along the red line were scored. The scale bar represents 50 μm.

Between the two paternal inbred lines, ZH71 exhibited a larger seed size and heavier weight than C72 during the process of seed development (Figure 1A; Supplementary Figure 1). The expression of *ZmARF12* in both inbred lines decreased after pollination; however, in compared with C72, ZH71 decreased more significantly at DAP10 stage (Supplementary Figure 15C). To further speculate whether the changes of seed weight and size were correlated with the expression level of *ZmARF12* in the different inbred lines, cell division was evaluated by examining the cell numbers per surface unit, and the number of cell layers in the grain pericarp at DAP10. The kernel pellicle of ZH71, which bore bigger kernels, had a smaller average cell size (larger cell size per unit), but more cell layers than those seen in C72, Z588, and Z58 (Figure 6).

**Figure 7:**
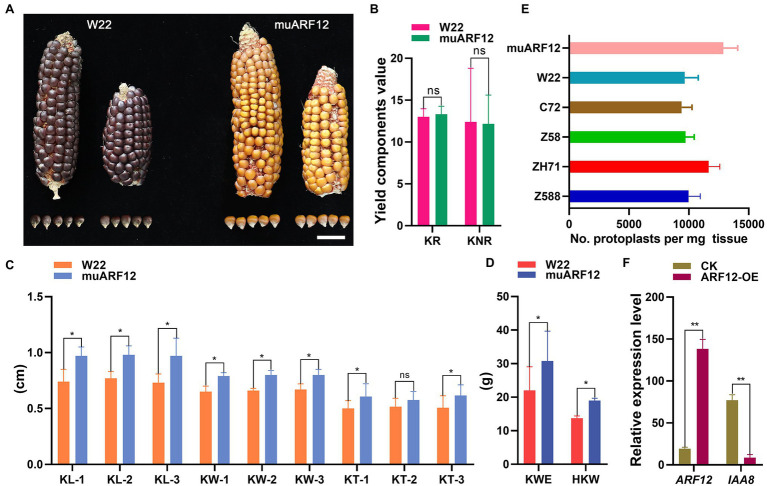
*Auxin Response Factor 12* of maize regulating cell division during kernel development. Comparison of seeds generated by wild-type and ZmARF12 mutants by evaluating the differences of phenotypes of the kernels **(A)** and the yield related traits **(B–D)**. Scale bar = 2 cm; KR, kernel rows; KNR, kernel number per row; KL, kernel length; KW, kernel weight; KT, kernel thickness; EW, ear weight; and HKW, hundred kernel weight. **(E)** Protoplasts yield of per mg tissues that isolated from the kernels of *ZmARF12* mutant, W22 and other four inbred lines at 10 days after pollination (DAP10). **(F)** The effects of ZmARF12 on the expression of ZmIAA8 were evaluated by transient over-expressing ZmARF12 in the protoplasts that isolated from maize kernels at DAP10. Each value is shown as the average of six independent experiments ± SD. ^*^p < 0.05, ^**^p < 0.01 (paired Student’s *t*-test); ns, not significant. Different letters on the columns show significant difference (*p* < 0.05) as determined by Tukey–Kramer test.

The five *ZmARF12* mutants (UFMu-04333, UFMu-09264, UFMu-09010, UFMu-09505, and UFMu-11213) obtained from Maize Genetics COOP were employed to elucidate the potential role of *ZmARF12* in regulating the kernel development process. Among the five mutants, UFMu-09010 exhibited larger kernel sizes (Figures 7A–C), higher KWE (kernel weight per ear), and HKW (hundred kernel weight, HKW) than those of wild-type W22 (Figure 7D). The protoplasts were isolated from the maize kernels of different inbred lines at DAP10 to further demonstrate the function and regulation of *ZmARF12*. The kernel meristem protoplast yield (per mg of tissue) was measured; the data showed that the muARF12 and ZH71 kernels yielded more protoplasts per mg tissue than other lines (Figure 7E). To test the regulatory influence of *ZmARF12* on the expression of *ZmIAA8*, the protoplasts were transfected with transient expression vector that harboring *ZmARF12* gene. The real-time RT-PCR was performed on the total RNA that extracted from the transfected protoplasts and un-transfected protoplasts as the CK experiment. The data indicated that *ZmARF12* was successfully transiently over-expressed in the transfected protoplasts, and that the expression of *ZmIAA8* in the protoplast cells transformed with *ZmARF12* was significantly lower than that observed in the CK data (Figure 7F). These data indicated that *ZmARF12* might be a regulator of cell division during seed development.

## Discussion

Examining intra-specific transcriptomic variations can provide insights into phenotypic variation. With the aid of transcriptome technology, we investigated the differential expression dynamics between different genotypes, and monitored the gene expression and regulatory patterns in hybrids during seed development.

The genes that were differentially expressed between the hybrids and their parents exhibited both additive and nonadditive expression patterns; the proportions of which differed between the studied hybrids and seven developmental stages. Several studies have reported high proportions of additive expression patterns in F_1_ hybrids (Jahnke et al., 2010; Hu et al., 2016). This was consistent with our observations that most of the DEGs (ZD958, Z58/ZH71, and Z588/C71) exhibited additive gene action at the start of pollination (DAP0). Nonadditive expression prevailed in the subsequent developmental stages of the three heterotic crosses, across the seven stages of ZD1002 seed development studied; in line with previous reports on maize seedlings, immature ears, primary roots, and embryos (Song and Messing 2003; Auger et al., 2005; Springer and Stupar, 2007; Hoecker et al., 2008). The shift from additive to nonadditive patterns between DAP0 and subsequent developmental stages was contrary to the results of many studies, as reviewed by Baranwal et al. (2012). In younger vegetative and reproductive stages, nonadditive expression accounted for major proportions of differential expression profiles (Baranwal et al., 2012). Differential expression activity has been reported to be significantly reduced, and additive expression as dominant, during the mature stages of vegetative development (Baranwal et al., 2012). Ko et al. (2016) found that temporal shifts in ZmCCA1-binding targets correlated with nonadditive and additive expressions during the early and late stages of seedling development. The shifts between additive and nonadditive expressions might be transitory, or a switch, to accelerate the rate of seed development in this work.

We noted additive and nonadditive DEGs were mainly affected by cis-only regulatory variation in more development stages for ZD1002 (Supplementary Figure 20). However, cis or cis coupled with trans × cis or other patterns mainly resulted in additive DEGs, whereas trans × cis largely affected nonadditive DEGs in ZD958 (Supplementary Figure 20). The results observed here were inconsistent with previous studies that trans-only regulatory variations were involved in nonadditive patterns, whereas cis-only patterns contributed to mid-parent expression (Zhou et al., 2019; Haas et al., 2020). Several investigations implicated that cis-regulatory variation in important roles for phenotypic evolution (Wittkopp and Kalay, 2012; Díaz-Valenzuela et al., 2020; Haas et al., 2020; Zhao et al., 2020). These changes of regulatory patterns were main drivers of the dynamic differences in seed size and weight between ZD1002 and ZD958.

Understanding the molecular mechanisms controlling seed development, seed size, and weight are important for improving maize seed yields. We constructed a set of co-expression modules with respect to the different developmental stages, and found 36 modules to be significantly and highly correlated modules with seed size and weight. Different modules recruited genes. Many genes were considered to be network hubs, with RP families and TFs comprising the two largest categories. RP-gene regulation of seed size has been reported in *Arabidopsis* and maize, identified through the analysis of mutants. Tian et al. (2017) reported that *NtRPL17* played an important role in establishing the size of embryos/seeds, by regulating cell cycle progression. In maize, emp5 mutants encode a PPR-DYW protein that is required for the editing of multiple transcripts in mitochondria, and the editing events, particularly the C-to-U editing at the rpl16-458 site, are critical to the mitochondrial functions and, hence, to seed development in maize (Liu et al., 2013). The *dek44* mutant produces small kernels with delayed development, and Dek44 encodes for the mitochondrial RPL9 (Qi et al., 2019).

Many of the TF families have been discovered to play roles in seed development (Bemer et al., 2010; Le et al., 2010; Agarwal et al., 2011). However, only a few TFs have been implicated in seed size and weight determination. Through WGCNA, Zhang et al. (2016) found that some ARF members were significantly correlated with seed weight, indicating their important roles in maize seed development. Congruently, several ARF genes were identified as being significant in our co-expression modules for seed size and weight. The seed size and grain weight of the *ZmARF12* mutant, UFMu-09010, were increased over those of the wild-type plants (Figures 7A–D). These observations were per mg tissue of the early stage fertilized seeds of the mutants, which yielded more protoplasts than other lines (Figure 7E). Overall, the expression of *ZmARF12* in the four inbred lines and their progenies exhibited a decreasing trend during the early stage of kernel fertilization (Supplementary Figure 15). The transcript levels of *ZmARF12* were negatively associated with cell division during kernel development (Figure 6). This was also confirmed by evaluating the yields of protoplasts that isolated from the kernels of other four inbred lines at DAP10 (Figure 7E). In addition, transient silencing of *ZmARF12* in maize Va35 plants facilitated cell expansion and division (Figure 5; Supplementary Figure 19), similar results were observed in the mutant UFMu-09010 (data not shown). The mutant phenotype and VIGS studies described here provide evidence that *ZmARF12* might repress cell division during kernel fertilization process, and have potential roles in determining the final seed size.

Auxin is a central plant hormone that regulates many processes, including pattern formation, cell division, and cell expansion (Chapman and Estelle, 2009; Vanneste and Friml, 2009; Weijers and Wagner, 2016); in which signaling is mediated by ARFs and Aux/IAA repressors that regulate the expression of a multitude of auxin response genes (Berleth et al., 2004). The transcript levels of ZmIAA8 increased within the first DAP8 and then decreased again in the following days of kernel fertilization (Supplementary Figure 15D), this initial increase of *ZmARF12* expression levels was also observed in the two maternal inbred lines, Z588 and Z58 (Supplementary Figure 15C). Mounting evidence indicates that ARFs regulate gene expression in response to auxin stimuli, and that ZmIAA8 is transcriptional repressor for auxin response genes (Ma and Dooner, 2010). The overlap between the ZmIAA8 distribution and *ZmARF12* expression levels showed that the regulatory role of *ZmARF12* might also depend on the auxin trends during the kernel development. During the early stages of fertilization, maize kernel development mainly depends on cell division (Guo et al., 2010; Huang et al., 2019). The transient silencing of ZmIAA8 impairs cell division (Figure 5), suggesting that during the early stages of seed development, *ZmARF12* might be part of the auxin-dominated regulatory mechanism that associated with cell division.

As their predominant mechanisms, ARFs bind to auxin response elements (AuxREs) as promoters of auxin-regulated genes, and to mediate auxin signal pathways by activating or repressing gene transcription. It is thought that auxin regulates ARF function through its effects on gene expression and protein turnover of Aux/IAAs. Aux/IAAs can dimerize with ARFs, and repress the ability of ARFs to activate gene expression in protoplast transfection assays (Schruff et al., 2006). In this work, transient over-expression of the *ZmARF12* in the protoplasts was shown to impair the expression of ZmIAA8 (Figure 7F). The mechanism by which this occurs remains obscure. Thus, the identification of the direct interaction of *ZmARF12* with its dimerization partners will help elucidate the underlying mechanism of *ZmARF12* function.

## Conclusion

To elucidate the underlying mechanism of maize seed development. Here, we report that transcriptional variations and network hub genes were involved in controlling seed size and weight through RNA-seq and weighted gene co-expression network analysis. The mutant phenotype and transient-silencing studies illustrated that *ZmARF12*, one of the network hubs, is a cell division repressor, and potentially determines the final seed size.

## Data Availability Statement

The original contributions presented in the study are publicly available. This data can be found here: National Center for Biotechnology Information (NCBI) BioProject database under accession numbers PRJNA728476 (DNA sequencing) and PRJNA649667 (transcriptome).

## Author Contributions

JM, ZF, YL, and YC designed the research, analyzed the data, and revised the final manuscript. YW and LN performed most experiments. BZ and XH helped with phenotype assessment. JC and XL helped with samples collection. JM and YC wrote the manuscript. All authors contributed to the article and approved the submitted version.

## Funding

This work was supported by the National Key Research and Development Program of China (2016YFD0300309/03), the special fund for Henan Agriculture Research System (S2010-02-G01), Major scientific and technological project of Henan province (151100111100), Fund for Distinguished Young Scholars from Henan Academy of Agricultural Sciences (2020JQ01), and Science-Technology Foundation for Outstanding Young Scientists of Henan Academy of Agricultural Sciences (2020YQ04).

## Conflict of Interest

The authors declare that the research was conducted in the absence of any commercial or financial relationships that could be construed as a potential conflict of interest.

## Publisher’s Note

All claims expressed in this article are solely those of the authors and do not necessarily represent those of their affiliated organizations, or those of the publisher, the editors and the reviewers. Any product that may be evaluated in this article, or claim that may be made by its manufacturer, is not guaranteed or endorsed by the publisher.

## Footnotes

^1^ ftp://ftp.ensemblgenomes.org/pub/release-40/plants/fasta/zea_mays/dna/

^2^ http://daehwankimlab.github.io/hisat2/

^3^ http://cole-trapnell-lab.github.io/cufflinks/install/

^4^ http://www.r-project.org/
