# Supplementary material for: Transcriptomic Variations and Network Hubs Controlling Seed Size and Weight During Maize Seed Development
Source: Front Plant Sci. 2022 Feb 14;13:828923. doi: 10.3389/fpls.2022.828923 (PMC8882617; doi:10.3389/fpls.2022.828923)
Supplement: Supplementary file 1 [file Data_Sheet_1.PDF]

# Transcriptomic variations and network hubs controlling seed size and weight during maize seed development

**Yanzhao Wang[1,†], Lihong Nie[2,†], Juan Ma[1,†], Bo Zhou[1], Xiaohua Han[1], Junling Cheng[1], Xiaomin Lu[1], Zaifeng Fan[3], Yuling Li[4], and Yanyong Cao[1,*]**

[1] Institute of Cereal Crops, Henan Provincial Key Laboratory of Maize Biology, Henan Academy of Agricultural Sciences, Zhengzhou, China, [2] Institute of Industrial Crops, Henan Academy of Agricultural Sciences, Zhengzhou, China, [3] State Kay Laboratory of Agro-biotechnology and Key Laboratory of Pest Monitoring and Green Management-MOA, China Agricultural University, Beijing, China, [4] Henan Maize Engineering Technology Joint Center, Henan Agricultural University, Zhengzhou, China

*** Correspondence:**

Yanyong Cao

yanyongcao@126.com

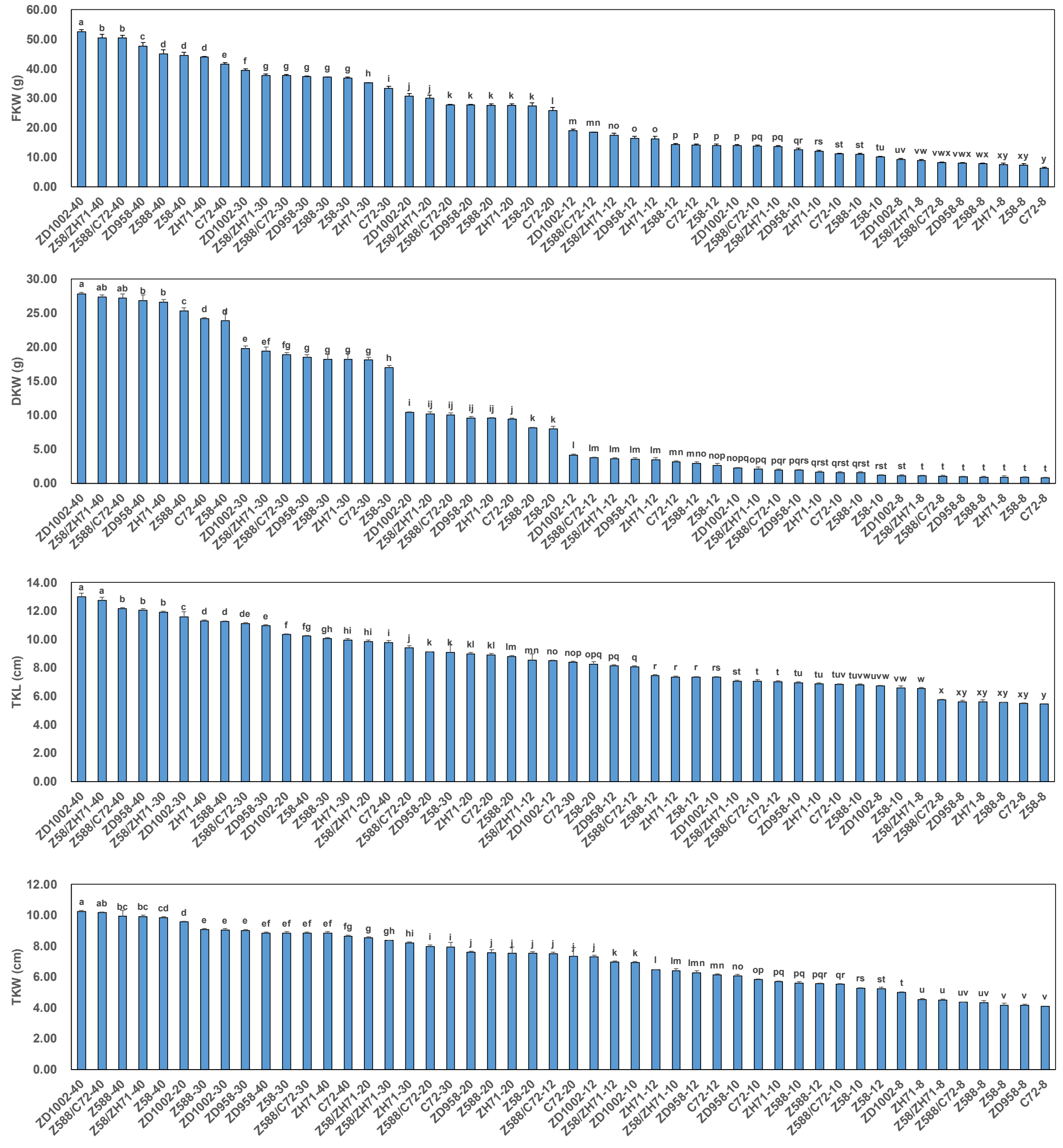


**Supplementary Figure 1.** Duncan's multiple comparisons of seed size and weight associated traits for eight genotypes during seed development. Data represent the means ± SD. Lower case letters above error bars represent results of Duncan's multiple comparisons at the 0.05 significant level. Means with different letters are significantly different. DAP8–40 represents 8–40 d after pollination, respectively. FKW, 100-fresh kernel weight; DKW, 100-dry kernel weight; TKL, Ten-kernel length; TKW, Ten-kernel width. Z588, Zheng588; Z58, Zheng58; ZH71, ZhengH71; C72, Chang7-2; ZD958, Zhengdan958; ZD1002, Zhengdan1002.

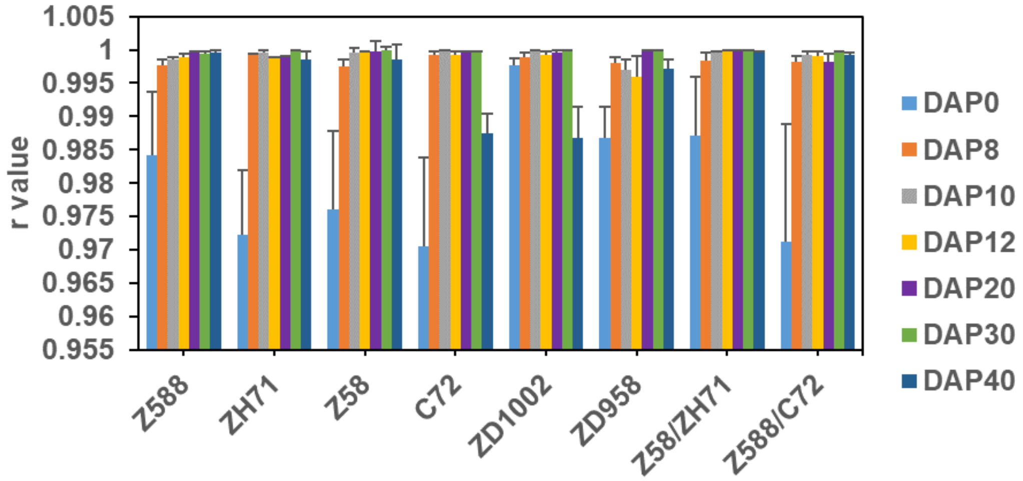


**Supplementary Figure 2.** The correlations of three biological replicates for eight genotypes at seven developmental stages. DAP0, DAP8, DAP10, DAP12, DAP20, DAP30, and DAP40 represent 0, 8, 10, 12, 20, 30, and 40 d after pollination, respectively. Error bars are represented by SD.

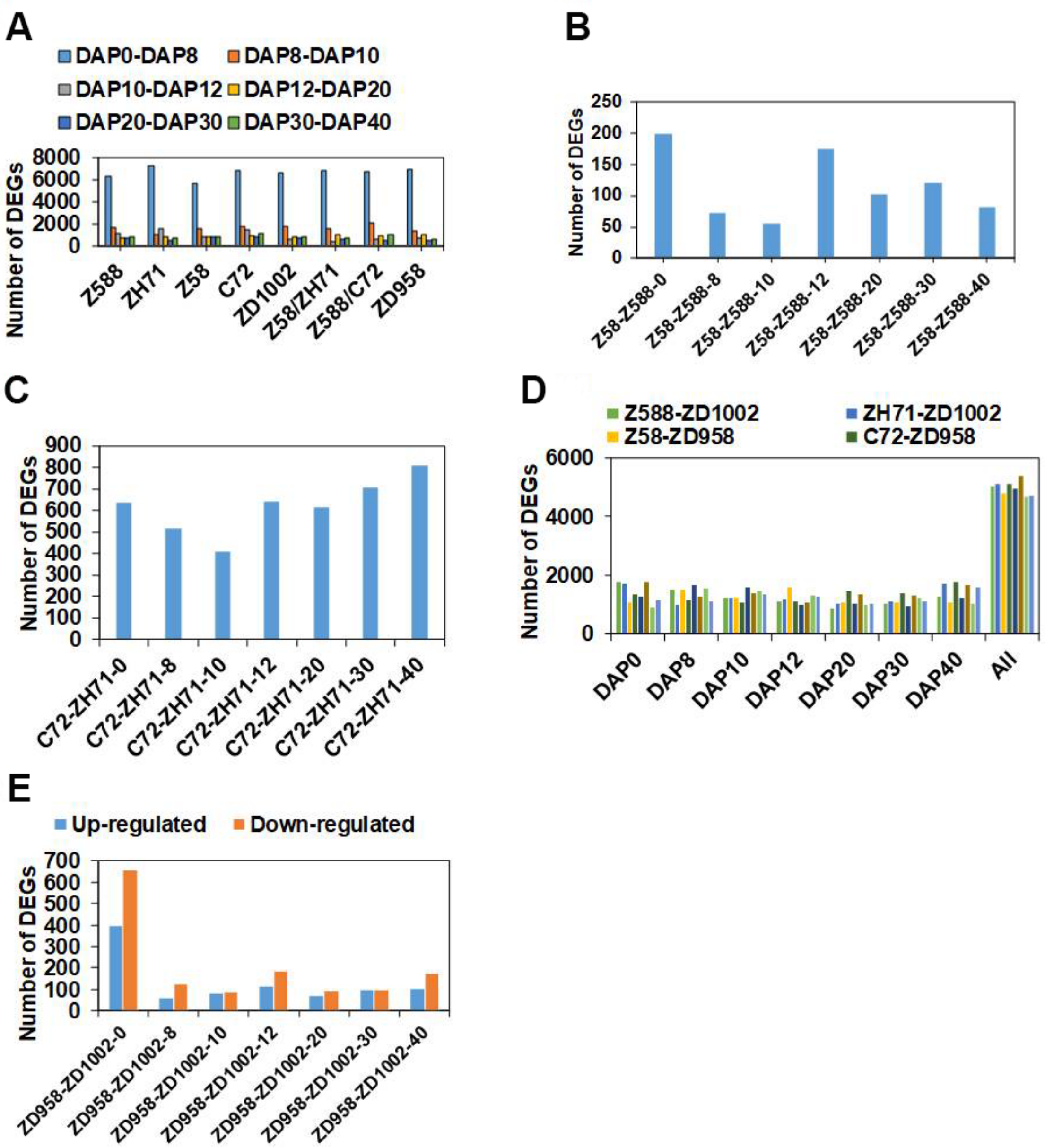


**Supplementary Figure 3.** Differentially expressed profiles. **(A)** The number of differentially expressed genes (DEGs) between two neighbouring stages. The previous stage is considered as CK. **(B)** The number of DEGs between Z58 and Z588. **(C)** The number of DEGs between C72 and ZH71. **(D)** The number of DEGs between $F_1$ and their respective parents (CK). **(E)** The number of up-regulated and down-regulated DEGs between ZD958 (CK) and ZD1002. 0, 8, 10, 12, 20, 30, and 40 denote 0–40 d after pollination (DAP). Z588, Zheng588; Z58, Zheng58; ZH71, ZhengH71; C72, Chang7-2; ZD958, Zhengdan958; ZD1002, Zhengdan1002.

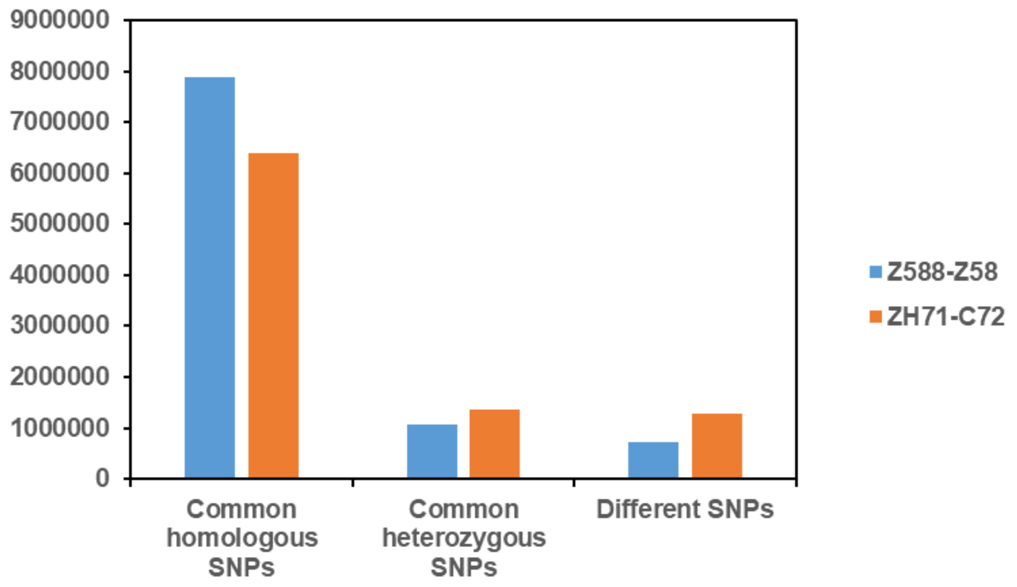


**Supplementary Figure 4.** The number of common and different SNPs between parents identified from DNA resequencing. Z588, Zheng588; Z58, Zheng58; ZH71, ZhengH71; C72, Chang7-2.

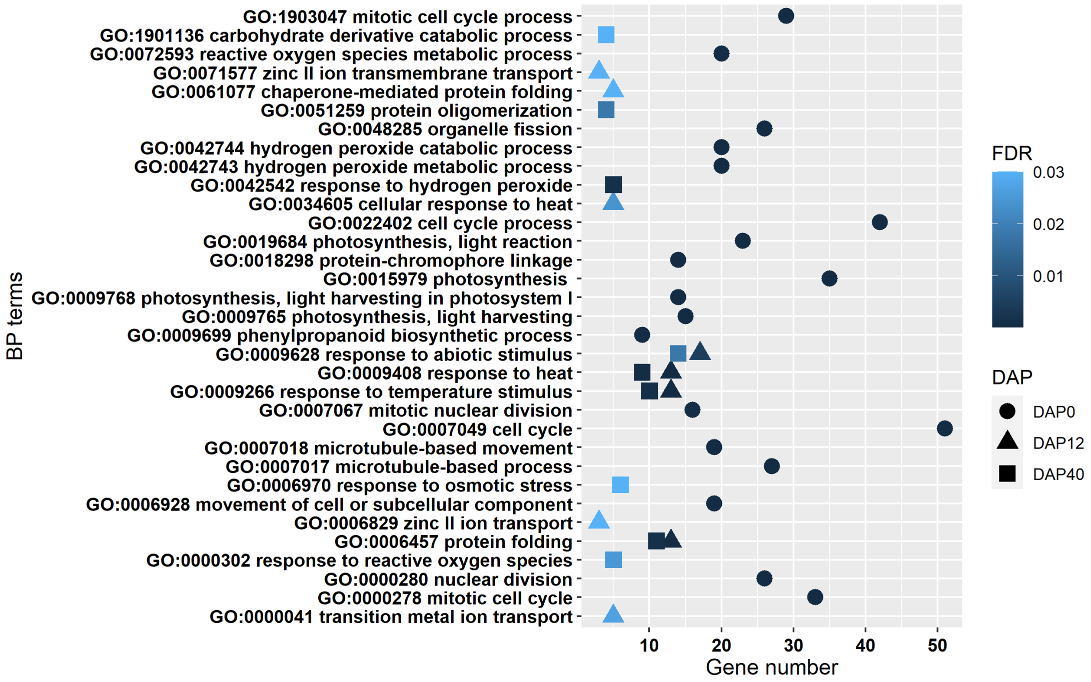


**Supplementary Figure 5.** Significant biological process (BP) terms of differentially expressed genes between Zhengdan1002 and Zhengdan958 at 0 d after pollination (DAP0), DAP12, and DAP40. Top20 terms are shown at DAP0 stage, and all significant BP terms are shown at DAP12 and DAP40 stages.

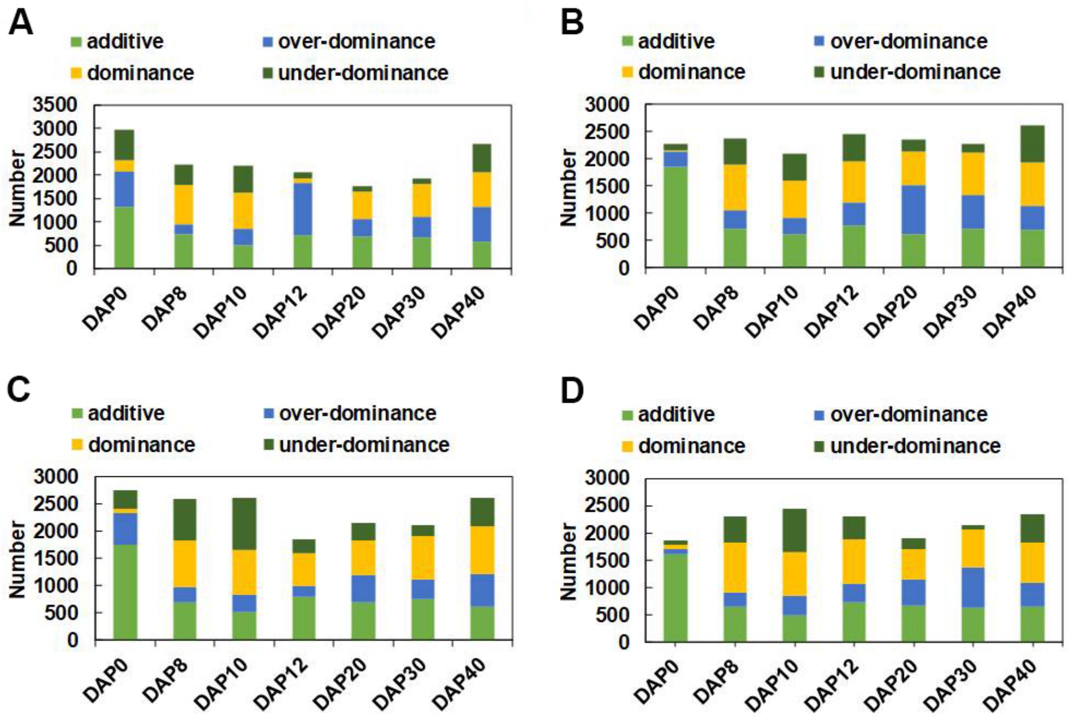


**Supplementary Figure 6.** Classification of additive and non-additive patterns in Zhengdan1002 **(A)**, Zhengdan958 **(B)**, Zheng588/Chang7-2 **(C)**, and Zheng58/ZhengH71 **(D)** at seven developmental stages. DAP0, DAP8, DAP10, DAP12, DAP20, DAP30, and DAP40 represent 0, 8, 10, 12, 20, 30, and 40 d after pollination, respectively.

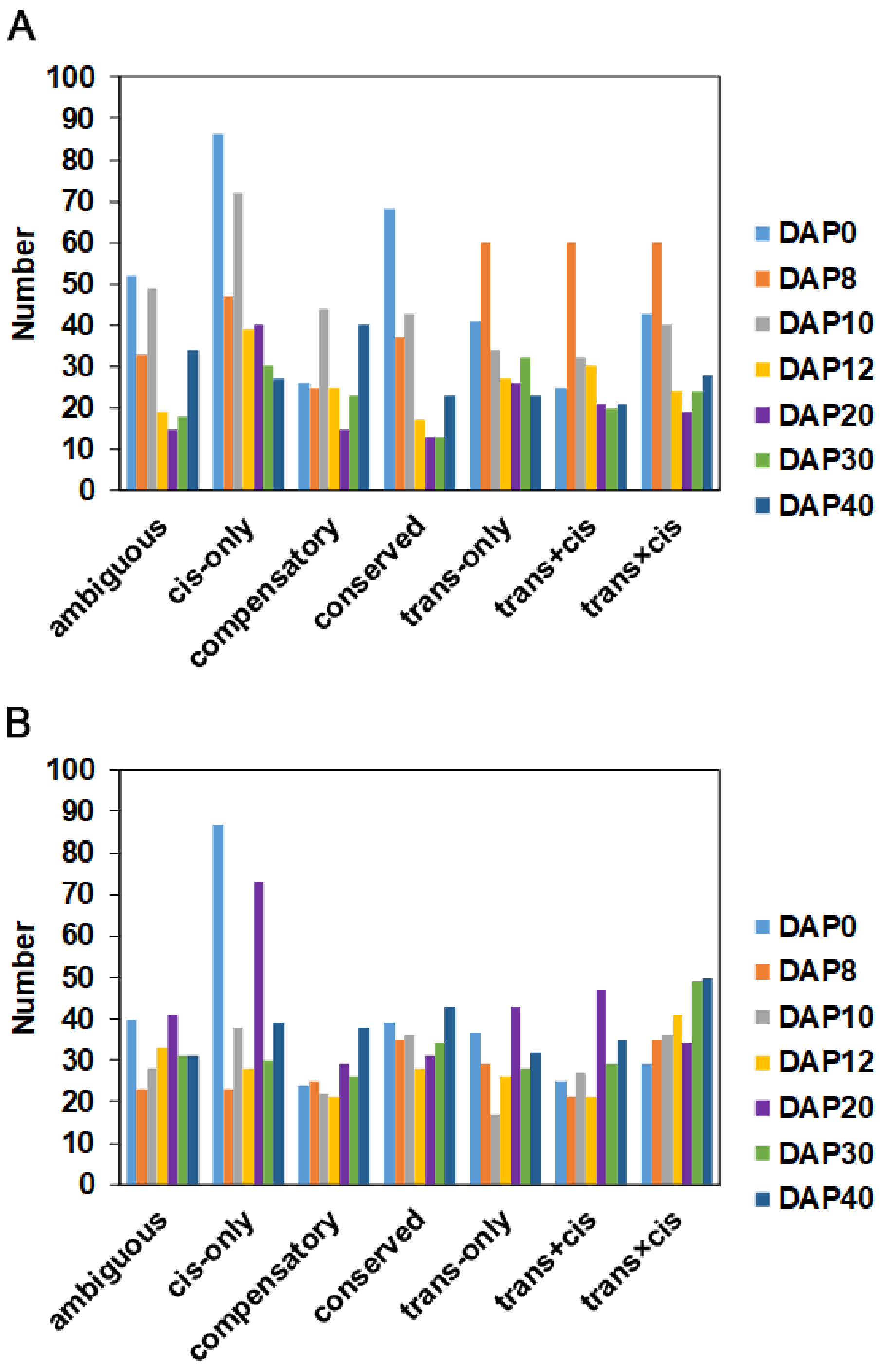


**Supplementary Figure 7.** Number of seven regulatory patterns at seven developmental stages in Zhengdan1002 **(A)** and Zhengdan958 **(B)**. DAP0, DAP8, DAP10, DAP12, DAP20, DAP30, and DAP40 represent 0, 8, 10, 12, 20, 30, and 40 d after pollination, respectively.

A

| | FKW | DKW | TKL | TKW |
|---|---|---|---|---|
| MEblue | 0.36 | 0.38 | 0.39 | 0.09 |
| MEmidnightblue | 0.17 | 0.17 | 0.13 | -0.31 |
| MEbrown | 0.35 | 0.36 | 0.22 | 0.06 |
| MEdarkorange | 0.19 | 0.15 | 0.14 | 0.24 |
| MElightgreen | 0.31 | 0.31 | 0.37 | 0.45* |
| MEpurple | 0.02 | -0.14 | 0.27 | 0.15 |
| MEdarkgrey | 0.05 | 0.06 | 0.07 | 0.46* |
| MEsaddlebrown | -0.34 | -0.52 ** | -0.10 | 0.08 |
| MEcyan | -0.11 | -0.13 | -0.23 | 0.16 |
| MEred | -0.24 | -0.26 | -0.39 | -0.10 |
| MEblack | 0.10 | 0.08 | -0.11 | -0.04 |
| MEdarkgreen | -0.09 | -0.11 | -0.27 | -0.38 |
| MEgrey | 0.07 | -0.08 | -0.05 | 0.02 |

B

| | FKW | DKW | TKL | TKW |
|---|---|---|---|---|
| MEsalmon | -0.04 | -0.10 | 0.09 | -0.07 |
| MEbrown | 0.06 | 0.11 | -0.09 | -0.05 |
| MEred | 0.30 | 0.35 | 0.13 | 0.24 |
| MEdarkorange | 0.02 | 0.08 | -0.07 | -0.23 |
| MEsteelblue | -0.27 | -0.25 | -0.25 | -0.49* |
| MEdarkturquoise | 0.28 | 0.27 | 0.22 | 0.48* |
| MElightyellow | 0.09 | 0.09 | 0.04 | 0.29 |
| MEroyalblue | 0.13 | 0.04 | 0.23 | 0.09 |
| MEdarkmagenta | 0.01 | -0.06 | 0.07 | -0.01 |
| MEdarkolivegreen | 0.32 | 0.26 | 0.29 | 0.27 |
| MEdarkgrey | -0.22 | -0.19 | -0.27 | -0.25 |
| MEblue | -0.39 | -0.41 | -0.30 | -0.39 |
| MEdarkred | -0.17 | -0.20 | -0.14 | -0.02 |
| MEgrey | 0.42* | 0.44* | 0.48* | 0.23 |

C

| | FKW | DKW | TKL | TKW |
|---|---|---|---|---|
| MEdarkolivegreen | 0.62** | 0.56** | 0.84** | 0.50* |
| MEfloralwhite | 0.48* | 0.39 | 0.60** | 0.45* |
| MEdarkgreen | 0.49* | 0.44* | 0.68** | 0.36 |
| MEdarkgrey | -0.04 | -0.03 | 0.36 | -0.20 |
| MEcyan | 0.26 | 0.23 | 0.42* | 0.20 |
| MEbrown4 | -0.21 | -0.25 | -0.30 | -0.10 |
| MEsaddlebrown | 0.28 | 0.14 | 0.31 | 0.32 |
| MEblue | -0.30 | -0.31 | -0.21 | -0.23 |
| MEorange | -0.80** | -0.77** | -0.65** | -0.71** |
| MEgreen | -0.16 | -0.18 | 0.15 | -0.21 |
| MEred | -0.33 | -0.36 | -0.10 | -0.34 |
| MElightsteelblue1 | -0.45* | -0.27 | -0.73** | -0.24 |
| MEorangered4 | -0.54** | -0.37 | -0.72** | -0.28 |
| MEsienna3 | -0.85** | -0.81** | -0.74** | -0.81** |
| MEpink | -0.20 | -0.23 | -0.08 | -0.24 |
| MEyellowgreen | -0.29 | -0.26 | -0.21 | -0.33 |
| MEgrey | 0.13 | 0.19 | 0.01 | 0.14 |

D

| | FKW | DKW | TKL | TKW |
|---|---|---|---|---|
| MElightyellow | -0.18 | -0.06 | -0.32 | -0.28 |
| MElightsteelblue1 | 0.28 | 0.45* | 0.33 | 0.30 |
| MEviolet | -0.04 | 0.23 | -0.07 | -0.09 |
| MEdarkmagenta | -0.41* | -0.76** | -0.51* | -0.41* |
| MEsteelblue | 0.07 | -0.28 | 0.11 | 0.16 |
| MEblue | -0.33 | -0.72** | -0.32 | -0.22 |
| MEdarkgrey | 0.07 | -0.23 | 0.15 | 0.22 |
| MEmidnightblue | 0.35 | 0.29 | 0.31 | 0.29 |
| MEdarkgreen | 0.37 | 0.64** | 0.35 | 0.27 |
| MEdarkorange | 0.06 | 0.21 | -0.04 | -0.08 |
| MEpurple | 0.60** | 0.89** | 0.67** | 0.57 |
| MEcyan | 0.54** | 0.55** | 0.67** | 0.64** |
| MEdarkturquoise | 0.49* | 0.52** | 0.59** | 0.53** |
| MEgrey | 0.07 | 0.07 | -0.04 | -0.13 |

E

| | FKW | DKW | TKL | TKW |
|---|---|---|---|---|
| MElightsteelblue1 | 0.29 | 0.11 | 0.19 | 0.39 |
| MEmediumpurple3 | -0.58** | -0.30 | -0.43* | -0.64** |
| MEmidnightblue | -0.46* | -0.25 | -0.47* | -0.46* |
| MEorangered4 | -0.32 | -0.13 | -0.19 | -0.33 |
| MEdarkorange | -0.04 | 0.27 | 0.23 | -0.14 |
| MEtan | 0.16 | 0.36 | 0.46* | 0.04 |
| MEdarkturquoise | 0.72** | 0.70** | 0.92** | 0.60** |
| MElightcyan1 | 0.39 | 0.61** | 0.71** | 0.24 |
| MEblack | 0.57** | 0.61** | 0.86** | 0.46* |
| MEdarkgreen | 0.18 | 0.40* | 0.63** | 0.08 |
| MEsienna3 | 0.42* | 0.25 | 0.47* | 0.37 |
| MEblue | -0.21 | -0.25 | -0.40 | -0.18 |
| MEdarkolivegreen | 0.33 | 0.33 | 0.35 | 0.25 |
| MEivory | 0.41* | 0.39 | 0.33 | 0.38 |
| MEgrey | 0.11 | 0.15 | 0.07 | 0.14 |

F

| | FKW | DKW | TKL | TKW |
|---|---|---|---|---|
| MEdarkmagenta | -0.38 | -0.23 | -0.24 | 0.03 |
| MEdarkolivegreen | -0.35 | 0.01 | -0.09 | -0.18 |
| MEsaddlebrown | -0.47* | -0.59** | -0.53** | 0.10 |
| MEbrown | -0.81** | -0.62** | -0.73** | -0.39 |
| MEgrey60 | -0.75** | -0.84** | -0.90** | -0.38 |
| MEplum1 | -0.74** | -0.87** | -0.88** | -0.18 |
| MEblue | -0.25 | -0.55** | -0.52** | -0.14 |
| MEdarkgreen | 0.38 | 0.01 | 0.15 | 0.39 |
| MEdarkgrey | -0.16 | -0.44* | -0.35 | 0.14 |
| MElightsteelblue1 | 0.23 | 0.02 | 0.12 | 0.37 |
| MEdarkorange | 0.72** | 0.58** | 0.70** | 0.56** |
| MEdarkturquoise | 0.19 | 0.20 | 0.26 | 0.35 |
| MEgrey | -0.11 | -0.08 | -0.21 | -0.18 |

**Supplementary Figure 8.** Module-trait relationship of the module eigengene correlation with seed size and weight at different development stages using WGCNA. **(A)**: 8 d after pollination (DAP8). **(B)**: DAP10. **(C)**: DAP12. **(D)**: DAP20. **(E)**: DAP30. **(F)**: DAP40. Modules are represented by colors. Shown are correlation values, and * and ** represent 0.05 and 0.01 significant level, respectively. FKW, 100-fresh kernel weight; DKW, 100-dry kernel weight; TKL, ten-kernel length; TKW, ten-kernel width.

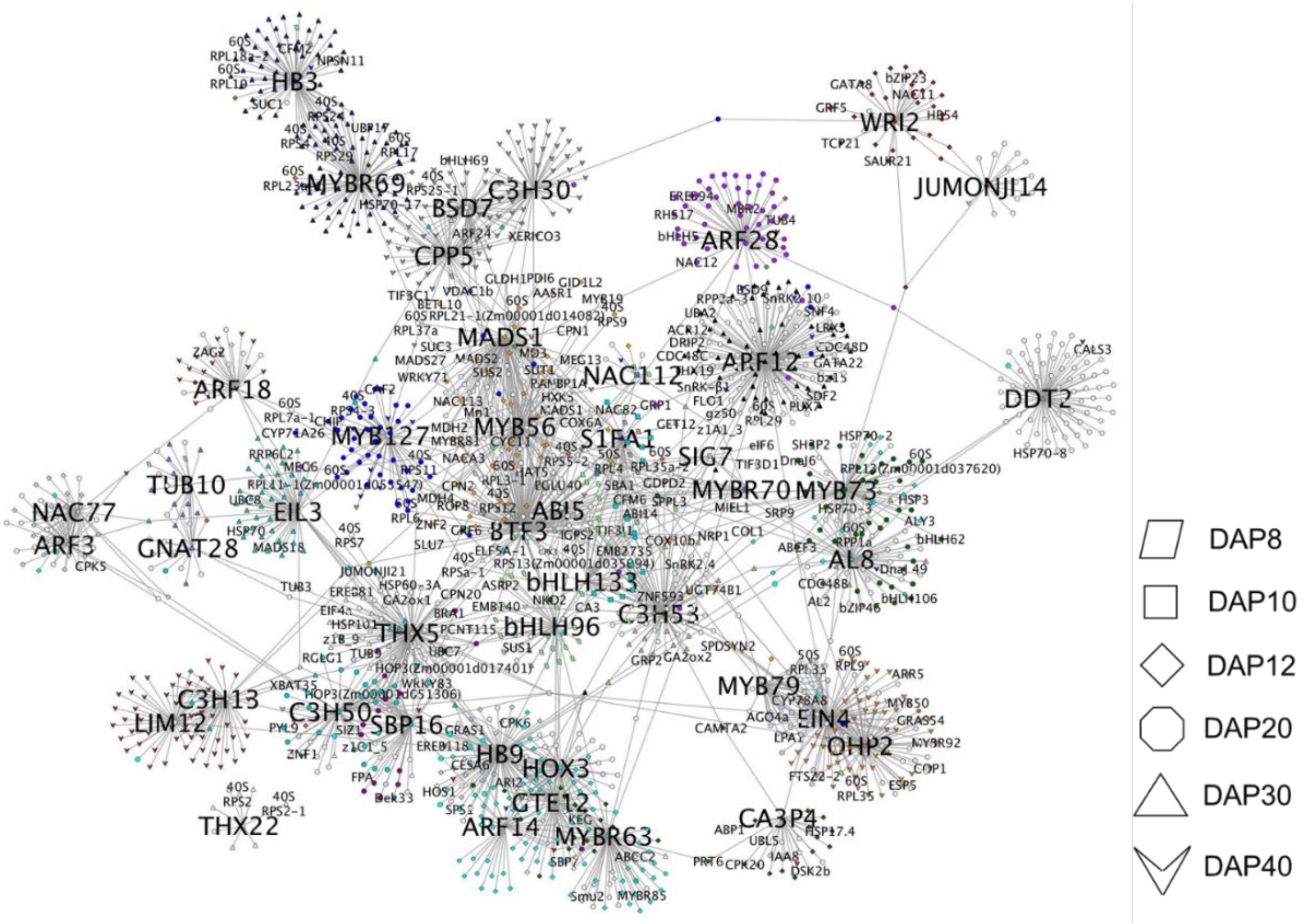


**Supplementary Figure 9.** Gene networks of hub genes associated with transcription factors at DAP8–40 stages. Top 10% genes are shown according their weight value with these hub genes. Different colors represent different module. Circle and geometric figures with no colors denote genes are appeared more than two stages or modules. DAP8–40 represents 8–40 d after pollination, respectively.

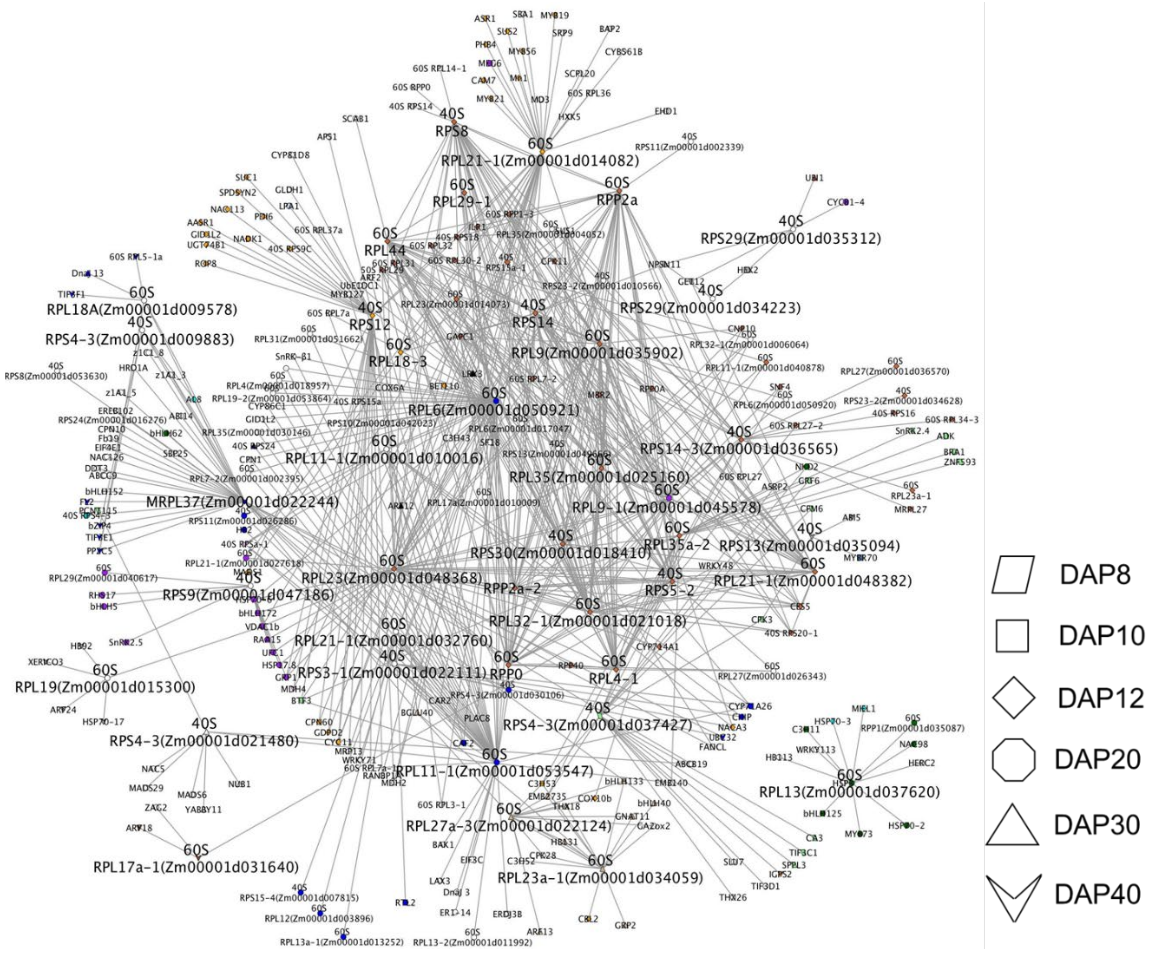


**Supplementary Figure 10.** Gene networks of hub genes associated with ribosomal proteins at DAP8–40 stages. Top 10% genes with annotations are shown according their weight value with these hub genes. Different colors represent different module. Circle and geometric figures with no colors denote genes are appeared more than two stages or modules. DAP8–40 represents 8–40 d after pollination, respectively.

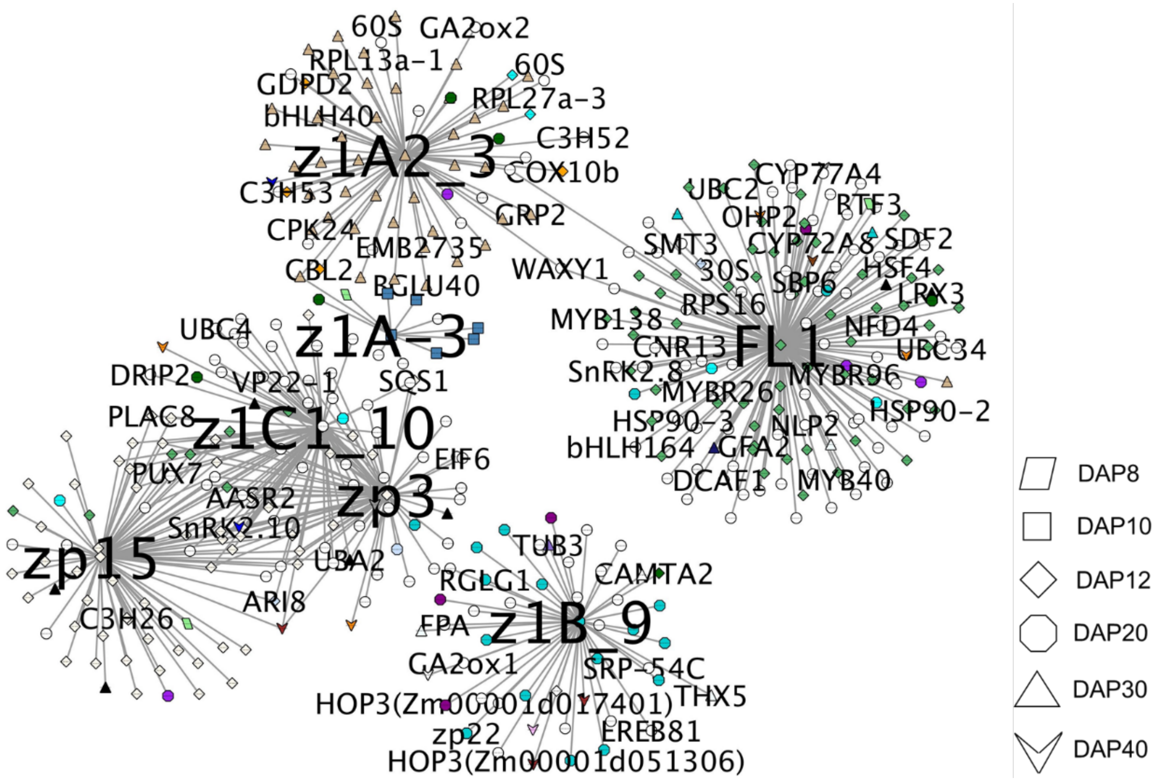


**Supplementary Figure 11.** Gene networks of hub zein genes at DAP8–40 stages. Top 10% of genes with weight value >= 0.1 are shown according their weight value with these hub genes. Different colors represent different module. Circle and geometric figures with no colors denote genes are appeared more than two stages or modules. DAP8–40 represents 8–40 d after pollination.

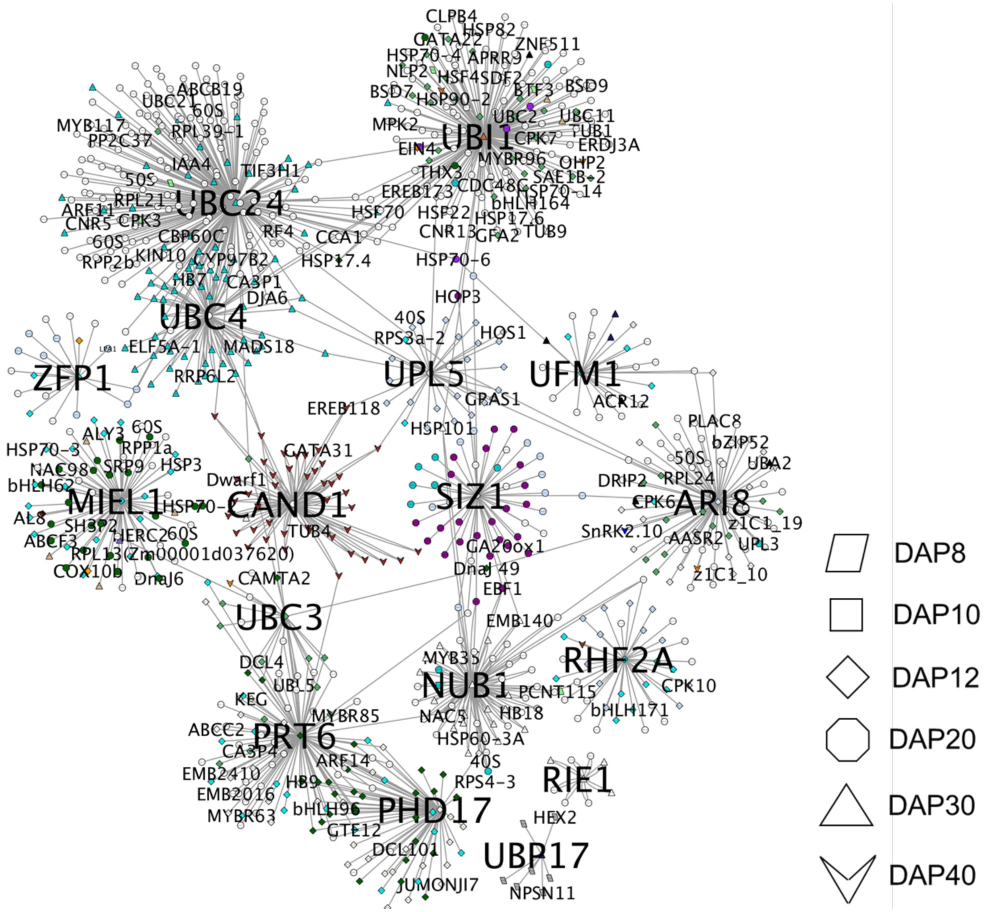


**Supplementary Figure 12.** Gene networks of hub genes associated with ubiquitin proteins at DAP8–40 stages. Top 10% of genes with weight value >= 0.1 are shown according their weight value with these hub genes. Different colors represent different module. Circle and geometric figures with no colors denote genes are appeared more than two stages or modules. DAP8–40 represents 8–40 d after pollination.

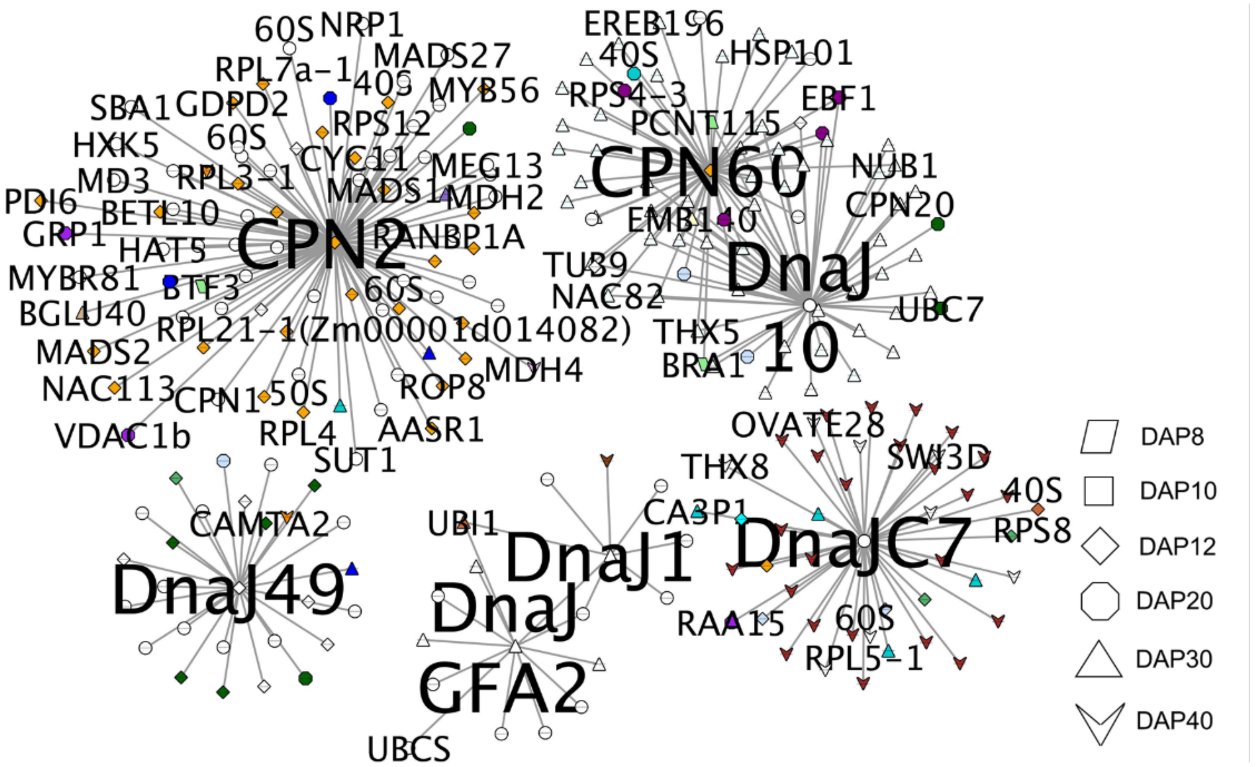


**Supplementary Figure 13.** Gene networks of hub genes associated with chaperone proteins at DAP8–40 stages. Top 10% of genes with weight value >= 0.1 are shown according their weight value with these hub genes. Different colors represent different module. Circle and geometric figures with no colors denote genes are appeared more than two stages or modules. DAP8–40 represents 8–40 d after pollination.

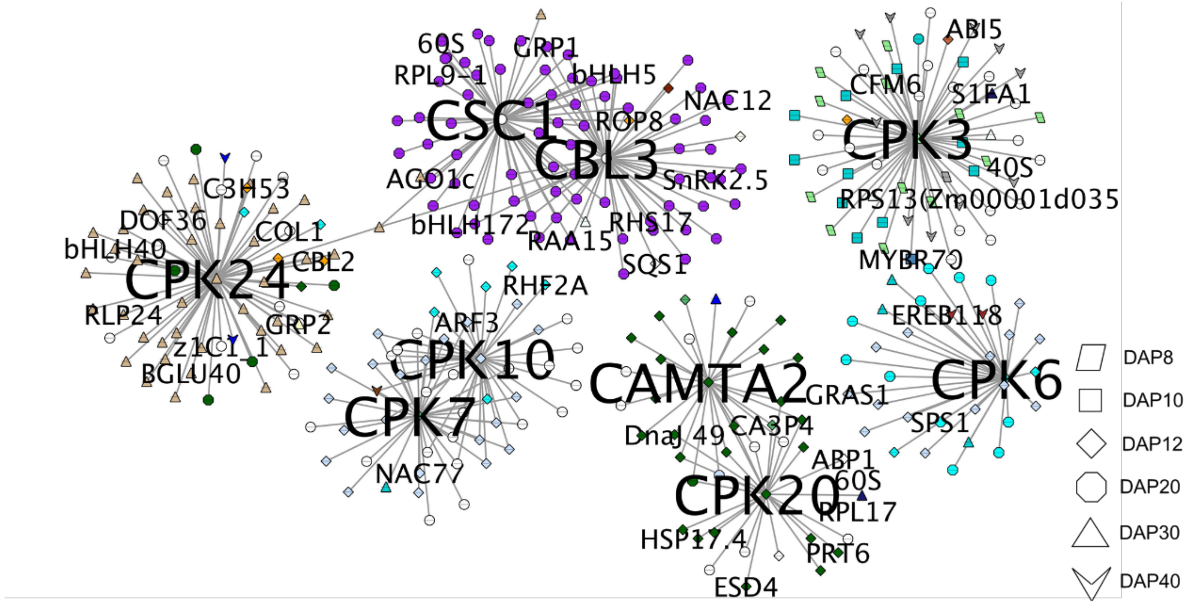


**Supplementary Figure 14.** Gene networks of hub genes associated with calcium-dependent protein kinases at DAP8–40 stages. Top 10% of genes with weight value >= 0.1 are shown according their weight value with these hub genes. Different colors represent different module. Circle and geometric figures with no colors denote genes are appeared more than two stages or modules. DAP8-40 represents 8–40 d after pollination.

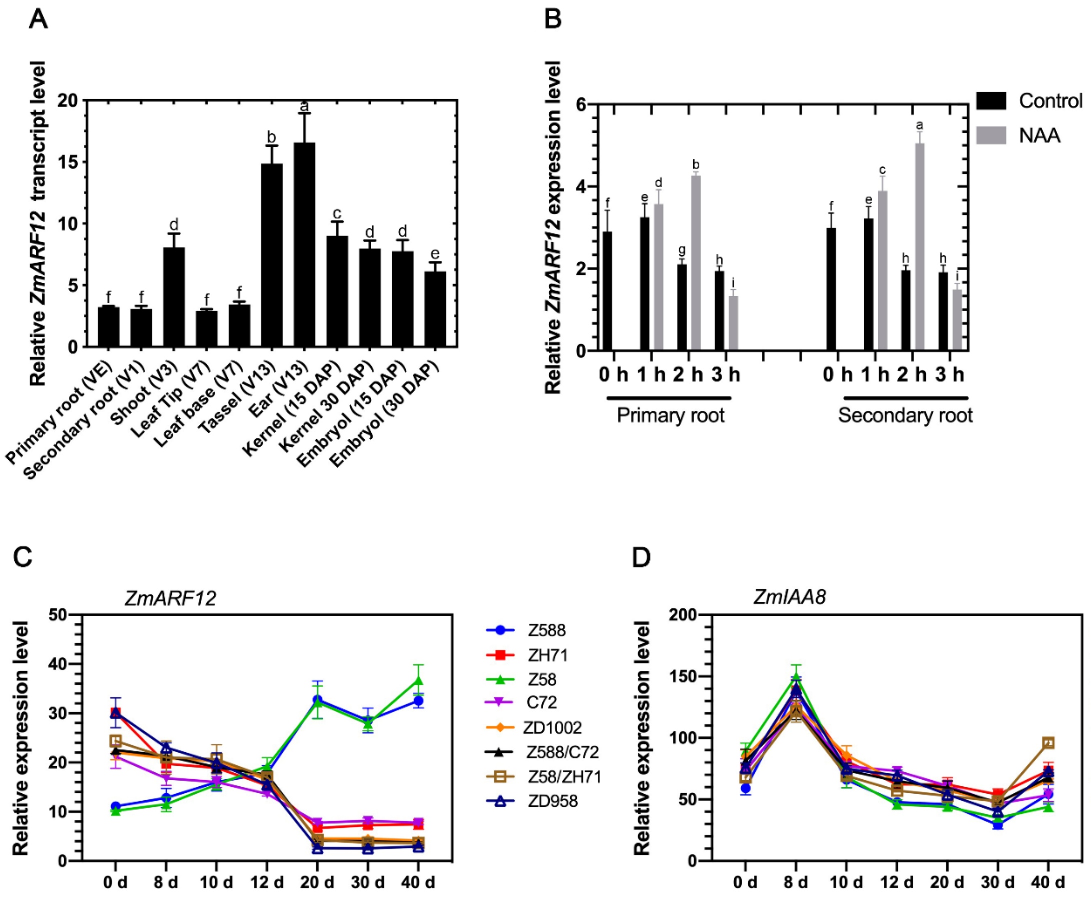


**Supplementary Figure 15.** The expression levels of *ZmARF12* and its potential interactor in different tissues, under auxin stimuli, and different time points post fertilization. **(A)** Real-time RT-PCR analysis of *ZmARF12* in different tissues. For each sample, the primary root and secondary root was cut from seedlings that had germinated for 8 d (vegetative emergency, VE) and V1 stage, respectively; the shoots were collected from plants at the V3 collar stage; the

leaf tip and leaf base were cut from plants at the V7 growth stage; and the tassel and ear were cut from plants at the V13 stage. Kernels were peeled off the ears at 15 d after pollination (DAP15) and DAP30, and the embryos were excised from the ears at 15 DAP and 30 DAP. **(B)** Fold change of *ZmARF12* conditioning exogenous auxin treatment. The primary and lateral roots of 8-day-old maize seedlings were isolated after 0 h, 1 h, 2 h and 3 h of incubation in 5 μM αNAA and Hoagland's solution control and subjected to real-time PCR analysis. **(C)** and **(D)** The expression profiles of *ZmARF12* and its potential interacting factor *ZmIAA8* during ear fertilization. The kernels of four hybrids (ZD1002, Zhengdan1002; Z588/C72, Zheng588/Chang7-2; Z58/ZH71, Zheng58/ZhengH71; ZD958, Zhengdan958) and their parental inbred lines (Z588, Zheng588; ZH71, ZhengH71; Z58, Zheng58; C72, Chang7-2) were harvested 0 d, 8 d, 10 d, 12 d, 20 d, 30 d, and 40 d after pollination and used for real-time RT-PCR. Relative mRNA abundance of each tested gene was averaged for triplicate reactions and the values were normalized according to the *Ct* of the internal control of ubiquitin gene by using the $2^{-\Delta\Delta Ct}$ method. All bars represent means ± SD (n = 6 repeats). Different letters indicate significant difference ($P < 0.05$) as determined by Tukey-Kramer test.

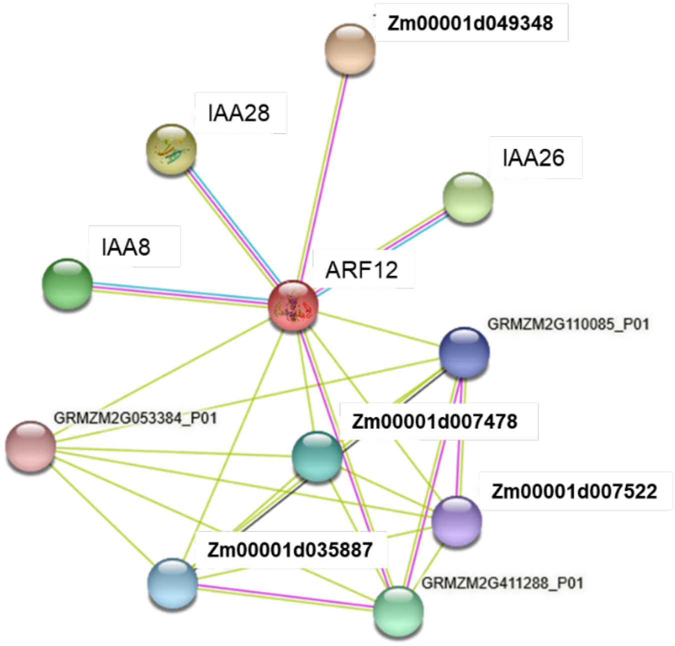


**Supplementary Figure 16.** Protein-protein interaction networks of ARF12 revealed by String database. The protein-protein interaction enrichment *P*-value was 5.21E-05.

```
ZmARF11  1    AGCAGCAGCTCACGAAACAGGAGCGTCATAAAGACATGGTCGTCGCAGGATAGCCTCAGACCCAGCGACGCAGCGGCAGCGACCCAGGAAGCAACAGCGA
ZmARF12  1    ----------------------------------------------------------------------------------------------------
ZmARF23  1    ----------------------------------------------------------------------------------------------------
ZmARF24  1    ----------------------------------------------------------------------------------------------------
ZmARF26  1    ---CGTAGTCCATAGTACTCCATTTGCA-AATTGCATTGCCACACC----TCGGCTCATCCCCACCT-CACCTCGGCCTCACTTCACAAGTTCACATCAC

ZmARF11  101  CCGGCATCGATCGCGGCAGCCGAGGCGGGGGACGGGAGGAGGGAGGGAGCGGTCGCCGCAGGAGCAGAGCGGCA-CGGCATGGGGTCCTGAGTGACTGA
ZmARF12  1    -------------------------------CTCCCCACCACTTCCCTCCCTTCC---CCGCCTACAAGCAAGCCGCCCCAGCCATTCATGGCCATCACC
ZmARF23  1    ----------------------------CGCAGGGGCGTGGGGACGGGACTGGCATTGCCACTGCCCAGCCGCGGGAGCGGCCCCTCCGGGGGA----
ZmARF24  1    ----TATAGGCCTCGGCCCCCATTCATGGCCATTACCACGATGTGTCACCCCACCACACCGCCCTCTCCGATGCTGCCTCCCTCATG-ATAACCCTCTCC
ZmARF26  91   ---GAAACAGGAGCGTCATAAAGACATGGGCGTCGCAGGGTAGCCTCAGACGGTCAGACCAGTGACGCAGCGGCAGCGGCAGCGGGCATGGGGTC-----

ZmARF11  200  CTGACTGACGACTGAGTGTCTGACTGAGAAGGAGATGGCTGGAGCGCGACGGCCCCGG-AGCCAT-TAATGGGGCGCTCATCCTCCTCGT-CCTCCCACT
ZmARF12  67   ACCA---------CCATGTGTCGCCGCCA-CTCCACCTCCACCTCCCACCCACTCCTCTCACCGCACCACTAAACCC--GCCTCCCTCGCTCCTCACCCG
ZmARF23  68   ------------CAAGCGCATTAATGCCACTGCCGCCTCCGCCGATTTAACTCCCCGC-CTCTAT-C------GCCCTCACCT---TCAC-CTGTACGCC
ZmARF24  96   CTGGTGGTTG-TTCTTTGCCTTGTTGCCG-TGCAGCCTCCACC-CCCACCCTCCTCATTAATCAC-TTGCTAGCTCCCTGCCTCCCTCCCGGCTCCCGCT
ZmARF26  183  -----------CTGAGTGACCGACTGCGAAGGAGATGGCTGGAGCGCGACGGCCCCGG-AGCCAT-TAATGGGGTGCTCATCCTCCTCCC-CCTCCT-CG

ZmARF11  297  CCTCGCCTCCCCCCCGCCA-GGTCGCGGTCGGCTCCGCTTTTAACCGGCTTGTCCTGGAGCGGGGCGAACGGCGAACCTCACGGGCGCCGGAACACGAA
ZmARF12  155  CTCCCTGTCCGTCCATCCATCGCCTCGCCGCCGCTCTGCTGCCGTTTG-CTCCAGCTGCGGC-CGACGGGGGCCATG------------------GCGGG
ZmARF23  145  GTCCCGCTTCGCTTCGGAGAGGTCGCTGCCTGTGCCTGCCATCAAC-GTTCCCTCGCTGGTTGAGGTAGACCC--------------------------
ZmARF24  192  CCCCCTTCTCGTGC-TTCGCGCCCCCGCAGCAGCCATGGCGGGGATCGACCTCAACGACACCGTGGAGGAGGACGAG------------------GAGGA
ZmARF26  269  CCTCCTCCCCGCCGGGTCGCGGACGCGGTCGGCTCCGCCTTTTAACCGGCCTGTCCGGGAGCGGGGCG--------------------------------

ZmARF11  396  CCGGAACCCTAGTGGTGGTGGTGGT----GGCGCGCCCCATGGGCATCGACCTCAACATGGCGGACGGCGAGAGCCAGGAGCGCCGCCCGCCGCCGGCGG
ZmARF12  235  CATCGACCTCAACACCGT--------GGAGGAGGAGGACGAGGAG-GAGGCGGAGGCGCTGCCTTTGCC--CGG--ACCCG--GTGGCCG--TGGCGCCG
ZmARF23  217  ACCGACCCCCAGTACTAGTCGGGAC----AGAG------ATGGGGATTGATCTCAACGCCGTGGG------------GGAGGACGACCCGGCGGGCGCGG
ZmARF24  273  GGCGGAGCCCGGCAACGCCTGCTCCAGCAGAGCCGGACCAGCTCCGCGGCCACGTTCCCGCCGCCGCCGCCGA--ACCAGCCGAGGCCGAGCGCCGCGG
ZmARF26  337  CCGGAACCCTAGTGCTGCTGGTGGCACGCGGCGCGCCCCATGGGCATCGATCTCAACATGGTGGACGGCGAGGGCCACGAGCGCCGCCCGCCGCCGGTGA

ZmARF11  492  TGTGCCGGAGCTGTGGCACGCGTGCGCGGGGCCCGTGGTTGCGCTACCGCGGCGGGGCAGCCTGGTCGTGTACCTGCCGCAAGGCCACCTAGCCGCGGC
ZmARF12  318  TGTGCCTGGAGCTGTGGCACGCGTGCGCGGGCCCCGTCCCGCCGCTGCCGCGGAAGGGCAGCGCCGTCGTGTACCTGCCGCAGGGCCACCTCGAGCACAT
ZmARF23  295  TGTGCGCGGAGCTGTGGCACGCGTGCGCGGGGCTGGAGTGGCGCTGCCGCGGCGGGGCAGCGCCGTGGTGTACCTGCCTCAGGCGCACCTCGCGGCGGG
ZmARF24  371  TGTGCCTCGAGCTGTGGCACGCCTGCGCCGGCCCCGTCGCGCCGCTGCCGAGGAAAGGGAGCGTCGTGGTGTACCTCCCGCAGGGACACATCGAGCACCT
ZmARF26  437  CGTGCCGGAGCTGTGGCACGCGTGCGCGGGGCCCGTGGTCGCGCTACCGCGCCGGGGCAGCCTGGTCGTGTACCTGCCGCAGGGCCACCTGGCCGCGGC
```

```
ZmARF11  592  CGGCGGCG--------------GCAACGTCGCTGTGGACC----------TGCCGCCGCACGTGGCGTGCCGCGTCGCGGATGTCGAGTTATGCGCGGAT
ZmARF12  418  CGGCGGCGACGCGGC-GC--GAGGAGCAGCAGCTTCGGCGGT---------GCCGCCGCACGTGCTCTGCCGCGTCGTCGACGTCACCCTCCACGCGGAC
ZmARF23  395  CGGCTGCGACGGTGG------TGGGGTGTCGGCGCCGGCCCCGCCGCGCGTGCCGCCGCACGTGGTGTGTCGCGTCGTCGACGTCGAGCTACGCGCGGAT
ZmARF24  471  CGGCGACGCCGCGGCCGCCGGCGGAGGCGCGCCGCCGCCCGTCGCCC---TGCCGCCCCACGTCTTCTGCCGCGTCGTCGACGTCACTCTCCATGCGGAC
ZmARF26  537  GGGCGGCG--------------GGGACGTCGCTGCGGACC----------TGCCGCCGCACGTGGTGTGCCGCGTCGCGGATGTCGAGCTATGCGCGGAT

ZmARF11  668  GCGGCGACGGACGAGGTGTACGCGCGGCTGGCGCTGGTCGCGGAGGGCGAGG----CATTTGGGAGAAATCTGCGTGGTGGTGGAGTCGATGGG--GACG
ZmARF12  506  GGCGCGACAGACGAGGTGTACGCGCGGGTGTCGCTGCTGCCCGAGGACGAGGACGCGGAGAAGCGGGCGCAGGCGCAGGCGCGGGTCCGGGAGGACGAGG
ZmARF23  489  GCGGCCACGGACGAGGTGTACGCGCGGCTTGCGCTGGTGGCGATGGATACGA----TGTTTGGCCGAAACATCAATGATGGTGAAACTGAAGAG--AAGA
ZmARF24  568  GCGTCCACGGACGAGGTGTACGCCCAGCTCGCCCTCGTCGCCGAGAACGAGGATGTCGCGAGGCGGCTGCGCGGACGGTCGGAGGACGGCAGCGCCGAGG
ZmARF26  613  GCGGCGACGGACGAGGTGTGCGCGCGGCTGGCGCTGGTTGCGGAGGGCGAGG----CATTCGGGAGGAATCTGGGCGGTGGTGGAGTTGAAGGG--GACG

ZmARF11  762  AT------GACATGGAGGATTTTGATGTTGAAAGGAAGTCC---CGGATGTTGCACATGTTCTGCAAAACGCTTACAGCCTCTGACACAAGCACACATGG
ZmARF12  606  ACCGCCGCGACGGCGAGGACGGCGGCGCCATGAGGCCGCTCGCACGGACGCCGCACATGTTCTGCAAGACGCTCACGGCATCCGATACCAGCACGCACGG
ZmARF23  583  AT------GGCGAGGAAGAGGATGGCGACGGAGAAAAGAAG---CACGCATCACACATGTTCTGCAAGACACTCACAGCTTCTGATACCAGCACACATGG
ZmARF24  668  AC------GGCGACGAAGGGGAAACCGTGAAGCAGCGGTTCTCGCGGATGCCGCACATGTTCTGCAAGACGCTCACGGCCTCCGACACCAGCACGCACGG
ZmARF26  707  AT------GGCATGGAAGATTTCGATGCTGAAAGGAAGTCC---GGGATGCTGCACATGTTCTGCAAAACGCTTACGGCCTCTGACACAAGCACGCATGG

ZmARF11  853  AGGGTTCTCTGTTCCTCGTCGTGCTGCTGAGGACTGTTTCCCGCCTCTGGATTATAATCAGCTCAGGCCTTCTCAAGAGCTGGTTGCCAAGGATTTGCAT
ZmARF12  706  CGGTTTCTCAGTGCCGCGCCGCGCCGCCGAGGACTGCTTCCCGCCGCTGGACTACAGCCAGCAGAGGCCGTCTCAGGAGCTCGTGGCCAAGGATCTACAC
ZmARF23  674  GGGATTCTCTGTTCCACGGCGAGCTGCAGAGGACTGCTTTCCACCATTGGACTATGAGCAGCTTAGGCCTTCCCAAGAGCTTATTGCCAAGGATTTGCAT
ZmARF24  762  CGGCTTCTCCGTGCCACGCCGCGCCGCCGAGGACTGCTTCCCGCCTCTGGACTACAGCCAGCAGCGACCGTCGCAGGAGCTTGTGGCCAAGGATTTGCAC
ZmARF26  798  AGGGTTCTCTGTTCCTCGCCGTGCTGCTGAGGACTGTTTCCCGCCTCTGGATTATAATCAGCTCAGGCCTTCTCAAGAGCTGGTTGCCAAGGATTTGCAT

ZmARF11  953  GGAGCCAAGTGGAAATTTCGTCATATATATAGGGGTCAGCCTCGTAGGCATCTCTTGACTACTGGATGGAGTTCATTTGTCAATAAAAAGAAACTGGTTT
ZmARF12  806  GGCACGGAGTGGAAGTTCCGGCATATCTATCGAGGTCAGCCACGAAGGCATCTCTTAACCACTGGATGGAGTGCATTTGTTAATAGGAAGAAGCTCATTT
ZmARF23  774  GGCATGAAATGGAGGTTCCGTCATATCTATAGAGGTCAACCTCGAAGGCATCTTCTGACAACTGGATGGAGTTCATTTATCAATAAGAAGAAACTAGTCT
ZmARF24  862  GGAACCGAGTGGAGGTTCCGCCACATTTATCGAGGGCAGCCCCGCAGACACCTTTTAACCACTGGATGGAGTGCCTTTGTCAACAAGAAGAAGCTTGTCT
ZmARF26  898  GGAGCCAAGTGGAAGTTTCGTCATATATATAGGGGTCAGCCTCGTAGGCATCTCTTGACTACTGGATGGAGTTCATTTGTCAATAAAAAGAAACTGGTTT

ZmARF11  1053 CTGGGGATGCGGTCTTATTTCTCCGAGGTGATGATGGTGAACTAAGGCTGGGCGTACGGAGGGCCATTCAGCTTAAAAATGAGGCCCTTTTTGACGATTT
ZmARF12  906  CTGGAGATGCAGTTCTCTTCCTTCGAGGCGAAGATGGAGTGCTTCGACTGGGAGTGCGCCGAGCAGCCCAGCTAAAAATTGTAACTCCTATTCCTGCACC
ZmARF23  874  CAGGGGATGCAGTCTTGTTTCTTAGAGGTAATGATGGTGAGCTAAGATTGGGTGTGAGGAGAGCAGTTCAACTGAAAAATGAAGCTCTACTTGAAGCTGT
ZmARF24  962  CAGGGGACGCCGTACTATTTTTGAGGGGTGATAATGGGGAGCTAAGACTTGGAGTGCGCCGTGCAGCTCAGCTTAAAAATGGATCTGCTTTTCCAGCTCT
ZmARF26  998  CAGGGGATGCTGTGTTATTTCTCCGAGGTGATGACGGTGAACTAAGGCTGGGTGTGCGGAGGGCTATTCAGCTTAAAAATGAGGCCCTTTTTGAAGATTT
```

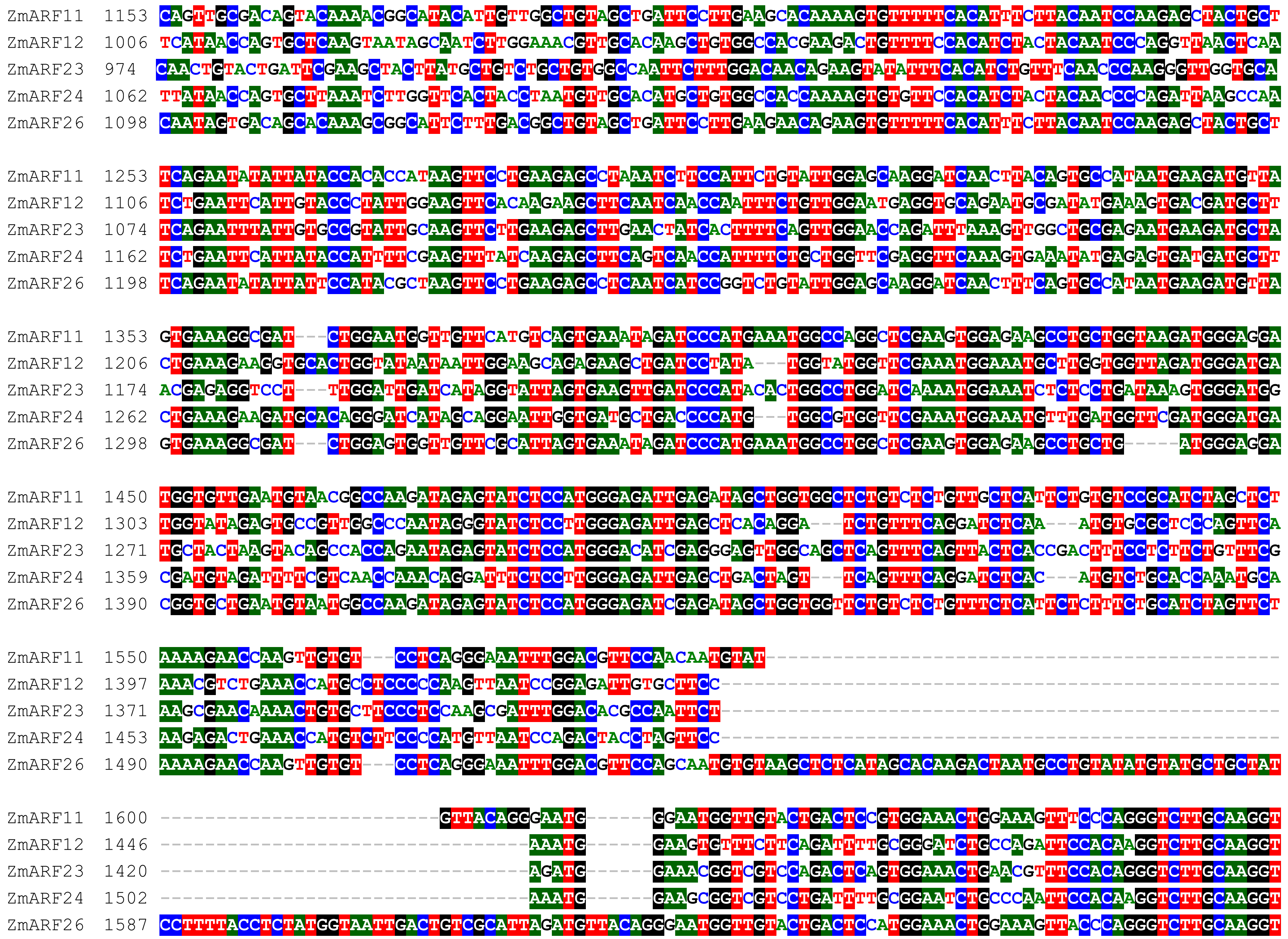

ZmARF11 1670 CAAGATTTGATGAGTTTTAGGACTCGTCA------TGTTCCGTCTGCTCCTCAAACTGTTGAGGCTGCAAAACTTCAATCTTCTGGTGCTAGTAGGTTCC
ZmARF12 1508 CAAGAATTGTTGGGTTTCAAAACCCATGATGGTCCTGCTATTTCTGCTTCTCAGGCAACTGAAGCAAGAAATTTGCAGTACAGTGATGAACG--------
ZmARF23 1482 CAAGAATTGGTGCACTCTAGTATTCATGG------TACTGCATGCTCTCATTCATCAGATAGCCCC---AGATGTCAAGGCTCTTATGGCAGGAGATTCT
ZmARF24 1564 CAAGAATTACTGGGTTATAGAACTCATGACAATGCTGCTGTTGCAACTTCTCAGCCATGCGAAGCAACGAACATGCAGTACATTGATGAACGAAGTTGCT
ZmARF26 1687 CAAGAATTGATGAGTTTTAGGACTTGTCA------TGTTCCGTGTGCTCCTCAAACTGCTGAGGCTGCAAAACTTCAATCTTCTGATGCTAGTAGGTTCC

ZmARF11 1764 TCAGTAATGCACGTGGCTGCGCATTGGGTGGTCCAACAAGCAGACTCGCAG---TGCATAA---CTCTGATTTTACCTACCAATCTGTAGGCTTCAATGA
ZmARF12 1599 ----------GAGTAACAATAACTTAGGGATCCCAAGACTTGGTGTTAGGTCTCCAACTGGAATCCCTGGGTTTCCCTACCATTGCTCAGGCTTTGGGGA
ZmARF23 1573 CTGCTGATGCGTGGAACTGCAAGATGAATGATGTAATGAGTGGGCCTCGACACCTAAATGC---CACTGGGTTTGCTTACCAGCCCCTAGGCTTCAGTGA
ZmARF24 1664 CCAACGATGCGAGTAACATTATCCCGGGGGTTCCAAGAATTGGTGTCAGAACACCACTCGGAAGCCCTAGGTTTTCCTACCGTTGCTCAGGCTTTGGGGA
ZmARF26 1781 TTAGTAATGCACATGGCTGCGCATTGGGTGGTCCAACAAGCAGACTCGCAG---TGCATAG---CTCTGGTTTTACCTACCAATGTGTAGGCTTCAATGA

ZmARF11 1858 ATCTATTGGATTCTCAGAGGTCTTGCAAGGTCAAGAAATTTCTCGGGCAGTTCCTATGTTCCAAGGAATGATGTCTGAGGCTTGTTCACTAAAAGGAGGA
ZmARF12 1690 ATCTCAAAGATTCCAAAAGGTCTTGCAAGGTCAAGAAGTGTTTCA------TCCTTTCCGAGGAGGATGTTTGGCTGATGGCCATATAAGAACTGCTGGC
ZmARF23 1670 ATCTGTCAAATTCTCAGAGGTCTTGCAAGGTCAAGAAATGTCTCAGGCGGTTCCTTCCTTCATGAGATCTGCTTTCAACTCTGGCACGCAGAATGGCAGG
ZmARF24 1764 GTCTCCAAGATTCCAAAAGGTCTTGCAAGGTCAAGAAGTATTTCA------TCCCTAC---AGAGGAACTCTGGTCGATGCAAGCTTGAGTAATAGTGGC
ZmARF26 1875 ATCTATTGGATTCTCAGAGGTCTTGCAAGGTCAAGAAATTTCTCGGGCGGTTCCTAAGTTCCAAGGAATGATGTCGGAAGCTTGTTCACTGAAAGACAGA

ZmARF11 1958 TATGGGCTGCATAGTTATATGCGTACCCCAGTTGCTGTTACTGGATTGTCAGCCACAACTCAAGAGTGTTCTCTCACACTATCTACTCC-GCCAGG----
ZmARF12 1784 ATGTATCAACCTGACGGTAGGCATGTATCTGGTGCAGCTTATAAATGGTCTGCAC---CACAAGGGTATGATTTCCCACAGCCAGCAAA-ACCGGTTTTT
ZmARF23 1770 GTTCGACCATTTGATTATGTGCAGAG---------------------ATCAGATGCAACTCAAGGATACGCTCTCCAGCAGTTTAATCT-GCCAGC----
ZmARF24 1855 TTCCATCAGCAAGATGGTTCTCATGTGCCTACTCAGGCCAGCAAGTGGCACGCACAGCTACATGGATGTGCTTTTC-GTGGCCAACAAGCACCAGCTGTT
ZmARF26 1975 TATGGGCTGCATAGTTATATGCGTGCCCCAGTTGCTGTTAATGGATTATCAGCTACAACTCAAGAATGTTCTCTCGCACTATCTACTCC-GCCAGG----

ZmARF11 2052 --AGCACAAGTTCCTTCTGTCTACCCTGATAATATTTTTAACCGAA------CTGTGGTTCGACAGCTTGGACTGGCAAGCAAGT---TTGATGGTGGAG
ZmARF12 1880 CTGTTGCAAGAATCATCCCCATCATCTGTGATGATGTTTCCACAAACTAGATCTAAGATAACTCACTTGGAATATGAGTACTC------AAGACATGAGG
ZmARF23 1843 --AACAGAAGTGCATTCGCCCTCTTCTGTTCTTATGTTTAACCAAA------CCATGGTACCACATGCTGAGCTAGATGGTGCGA---CCAACCGTGAAG
ZmARF24 1954 CCATCTCAATCCTCATCCCCACCATCTGTCCTGATGTTTCAACGAGGTGATCCAAAGATGTCCCCATTTGAATTTGGGCATTTCCACGTGAATAAGAAAG
ZmARF26 2069 --AGCACAAGTGCCCTCTCTCTACCCTGGTAATGTTGTTAACCGAA------CTGTGGTTCCACAACTTGGACTGGCAAGGAAGT---TTGGTGGTGGAG

ZmARF11 2142 CTA-CAAATGCCCAGCAGTCTGTCCCATTTGATAGGCCGAGGGAA--ATTTGGAGCA-AGCCA------CAGCATGAAATATCTGAT-CAAACGAAAATG
ZmARF12 1974 ATGGTAGACTTGATAGGACTGTTCCTACTCAAGACATGGAAGAAGCAATCAAACATTATCTCTTTG-GCCTCATCTTGTTTCAGGC-GAAGCAATAGAA
ZmARF23 1933 AAG-TACATGGCAGCAGGTACTTGTCATCCAATGCAATAGGGAGAGAAGCTGAACCATGGCCATCCATGCAGCAGCAAAGAGCGAGTGTAAATGGAAGCG
ZmARF24 2054 AGGATAGACGCGCAATGTTTGTCCATGCTGGAGGCATCGGAGGAACTGAGCAAACGACGATGCTCCA-GGCTCATCATGTTTCTGGA-GGAACGGGAAAC
ZmARF26 2159 GTA-CAAATGGCCAGCAGTCTGGCCCATTTGATAGGCCGAGGGAA--CTTTGGACCA-GGCCA------CAGCATGAAACACCTGAC-CAAATGAACTTG

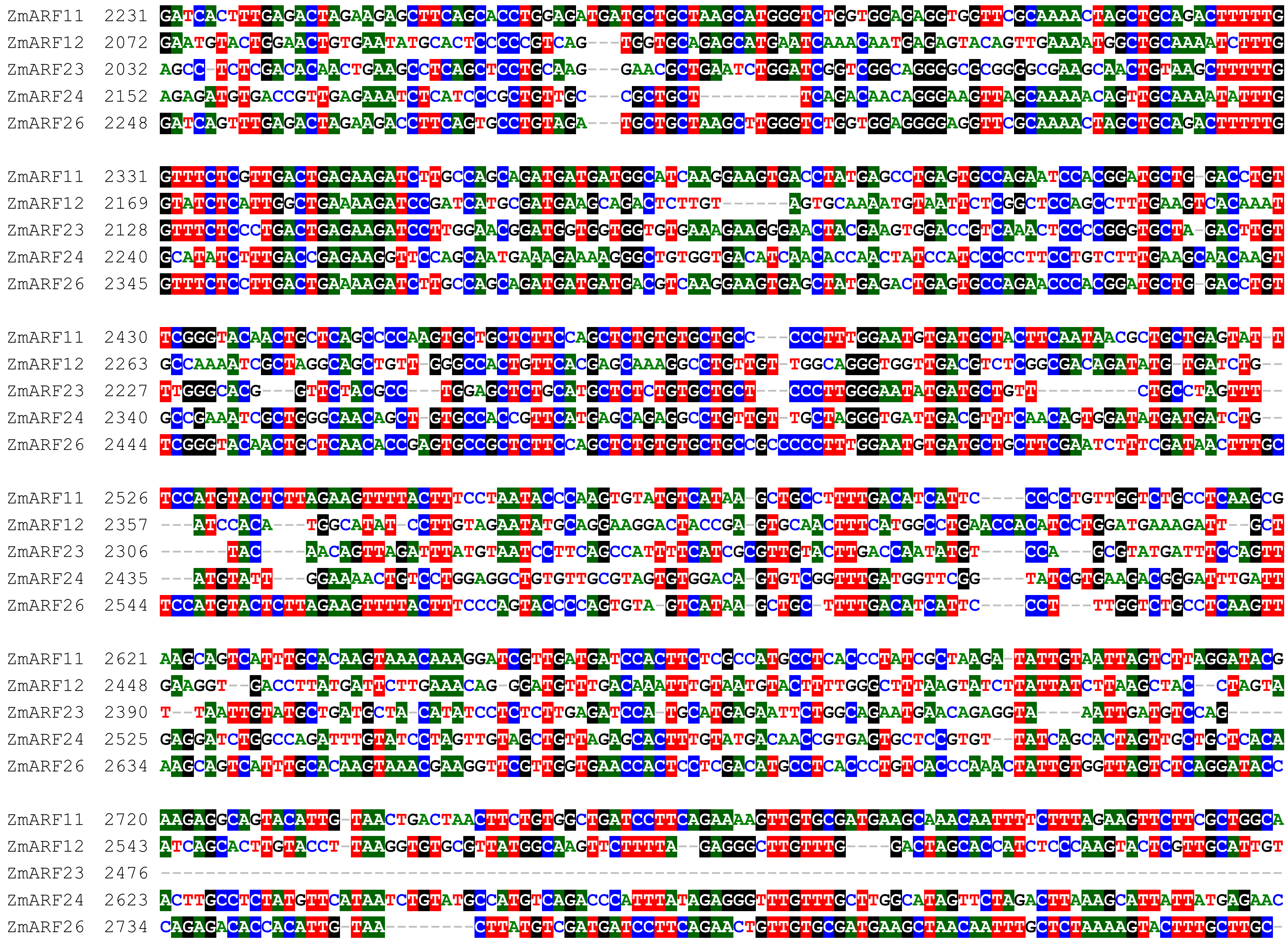
ZmARF11 2231
ZmARF12 2072
ZmARF23 2032
ZmARF24 2152
ZmARF26 2248
ZmARF11 2331
ZmARF12 2169
ZmARF23 2128
ZmARF24 2240
ZmARF26 2345
ZmARF11 2430
ZmARF12 2263
ZmARF23 2227
ZmARF24 2340
ZmARF26 2444
ZmARF11 2526
ZmARF12 2357
ZmARF23 2306
ZmARF24 2435
ZmARF26 2544
ZmARF11 2621
ZmARF12 2448
ZmARF23 2390
ZmARF24 2525
ZmARF26 2634
ZmARF11 2720
ZmARF12 2543
ZmARF23 2476
ZmARF24 2623
ZmARF26 2734

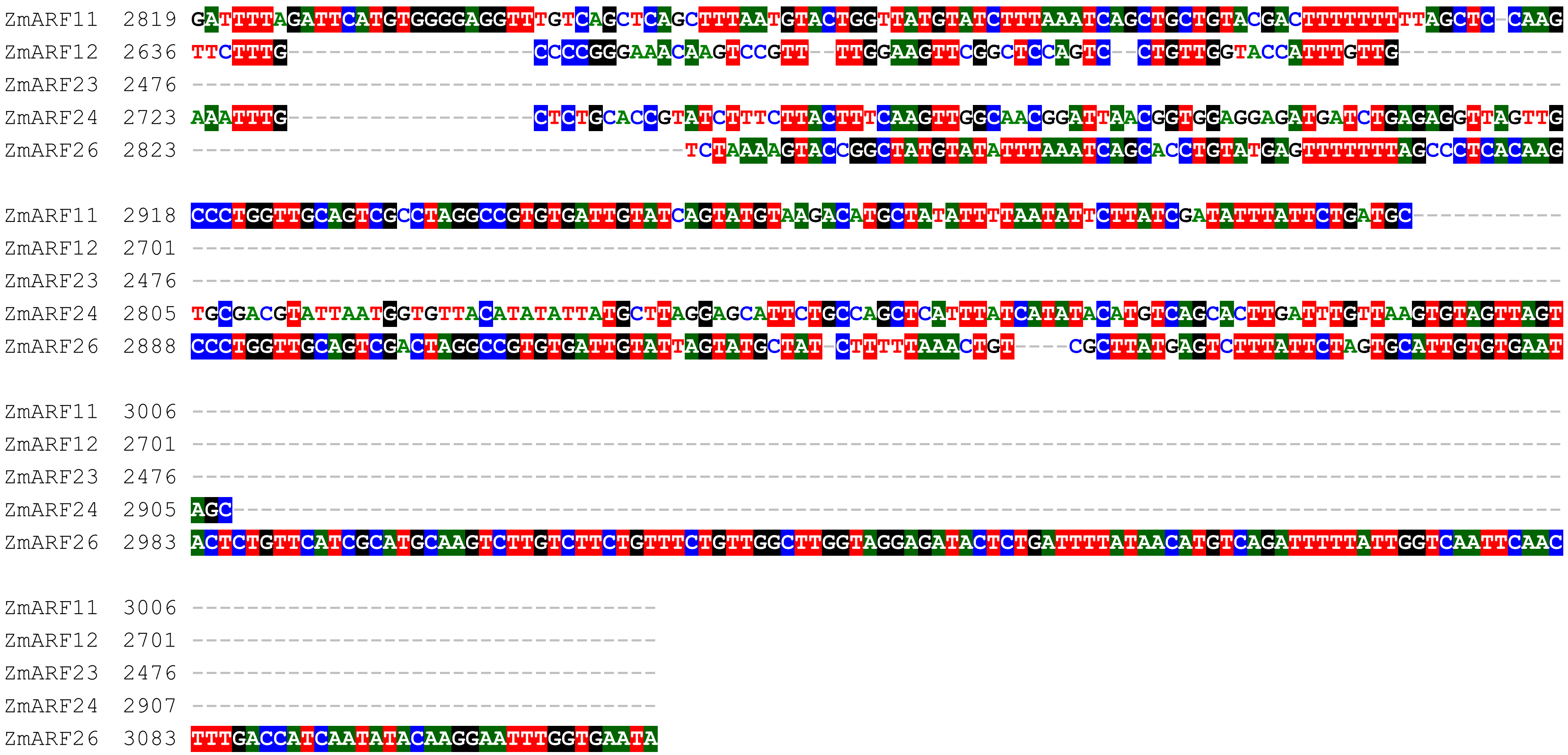

**Supplementary Figure 17.** The alignment of different *ZmARF* orthologues. The alignment was performed using BioEdit (v7.2.5, http://www.mbio.ncsu.edu/bioedit/bioedit.html). Sequences of *ZmARF* genes analyzed in the alignment were obtained from MaizeGDB. Accessions were as follows: *ZmARF11*, GRMZM2G056120; *ZmARF12,* GRMZM2G437460; *ZmARF23*, GRMZM2G441325; *ZmARF24,* GRMZM2G030710*; and ZmARF26,* GRMZM5G874163.

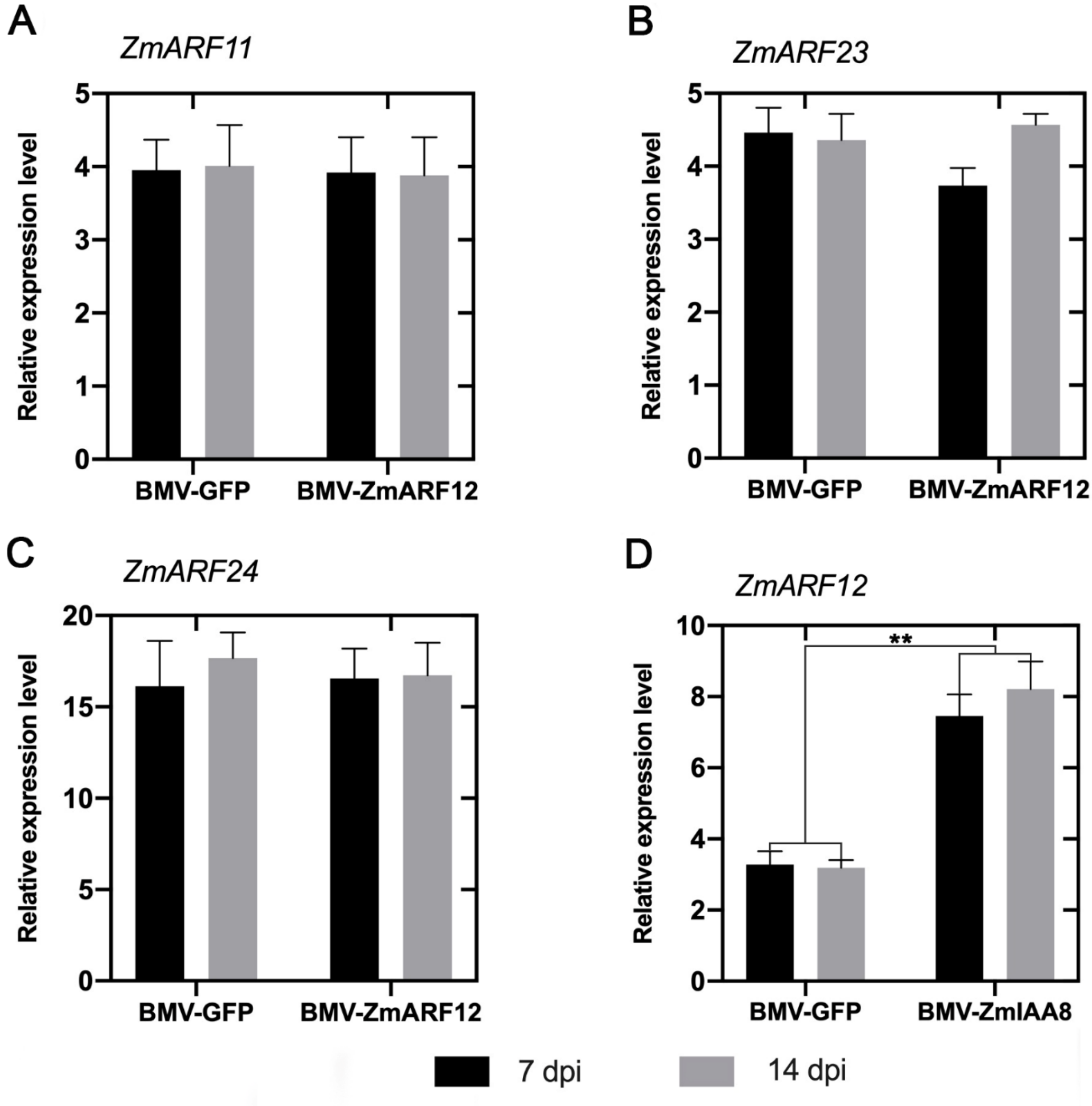


**Supplementary Figure 18.** The expression level of *ZmARF11*, *ZmARF12*, *ZmARF23*, and *ZmARF24* in *ZmARF12*- and *ZmIAA8*-silenced maize seedlings. **(A)** to **(C)** The silencing specificity of *ZmARF12* was evaluated by detecting the expression levels of *ZmARF11*, *ZmARF23* and *ZmARF24*, which share high identities with *ZmARF12*, in the relevant systemic leaves using qRT-PCR at 7 days and 14 days post inoculation (dpi), respectively. **(D)** The relative transcript level of *ZmARF12* in the *ZmIAA8* transient knocked-down plants was evaluated by using real-time RT-PCR at 7 dpi and 14 dpi, respectively. Values are means ± SD, * $P < 0.05$, ** $P < 0.01$ (paired Student's *t*-test); ns, not significant.

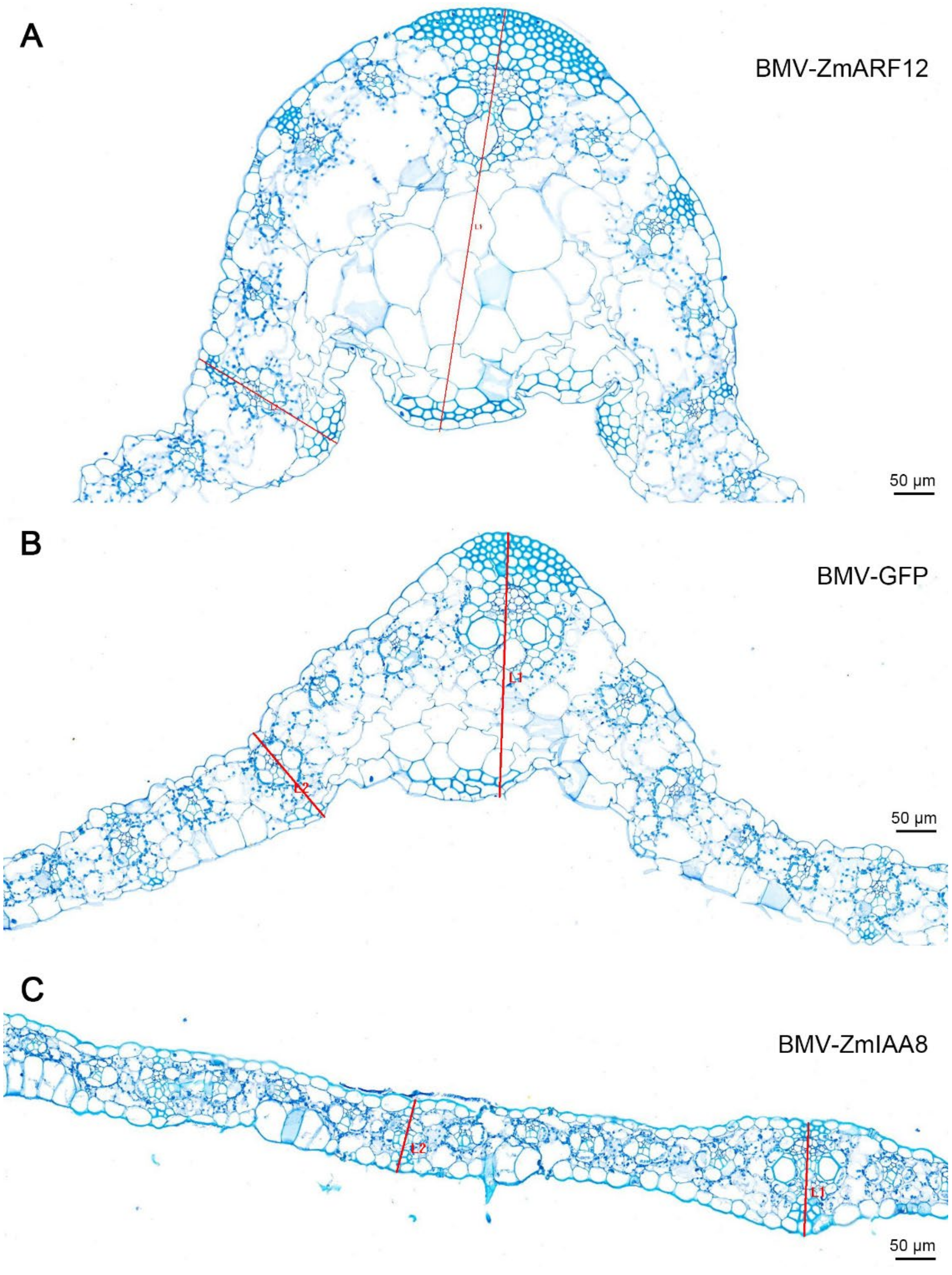


**Supplementary Figure 19.** Microscopic analysis of the leaf disk of the *ZmARF12*- and *ZmIAA8*-silenced plants. The leaf disks were dissected from the second systemic leaves above the inoculated leaves of the *ZmARF12* transient silencing, *ZmIAA8*-silenced and control plants, respectively. The cells along the two red lines (L1 and L2) were calculated, respectively. The scale bar = 50 μm.

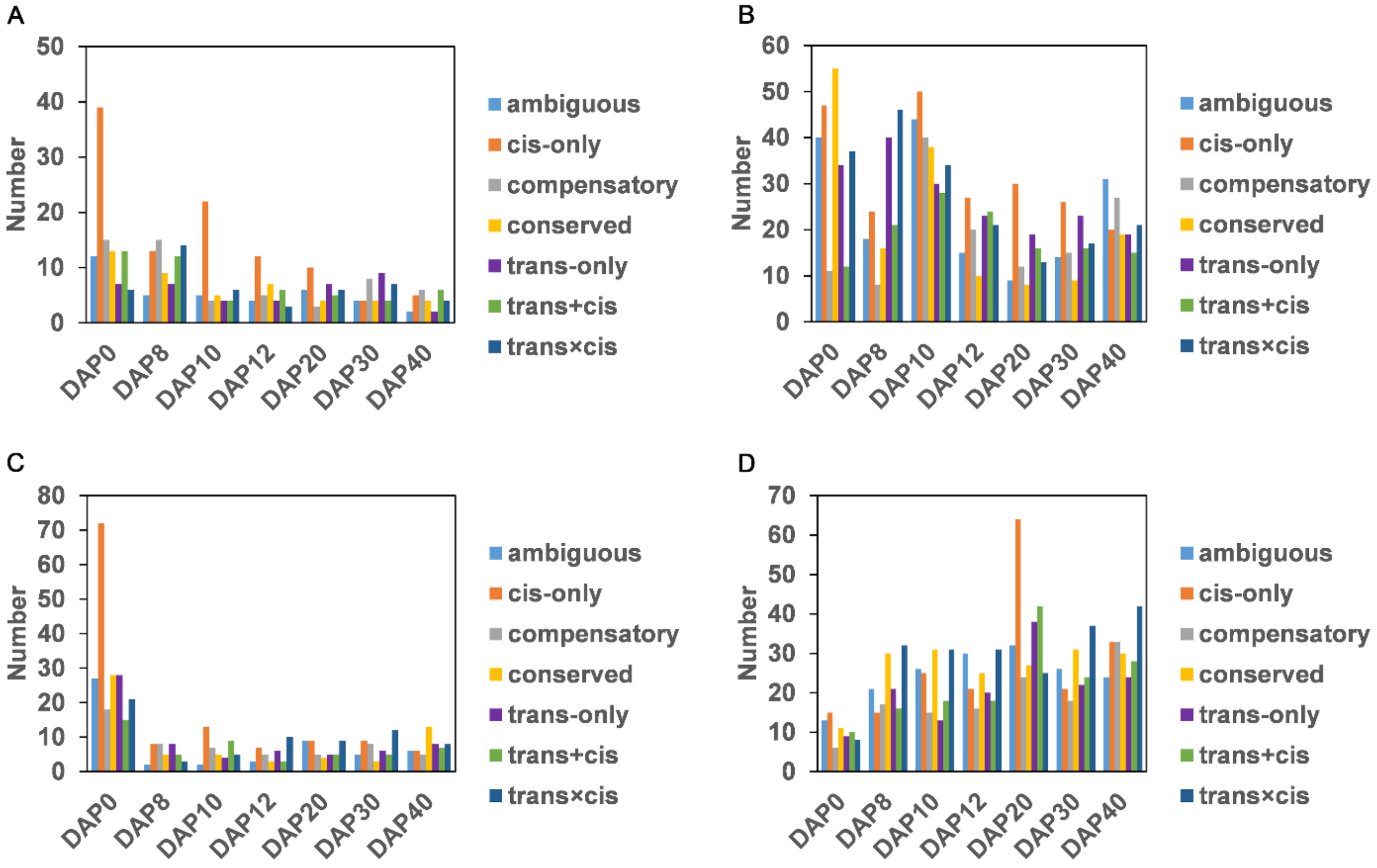


**Supplementary Figure 20.** Regulatory category assignments of additive **(A)**, **(C)** and nonadditive **(B)**, **(D)** differentially expressed genes in Zhengdan1002 **(A)**, **(B)** and Zhengdan958 **(C)**, **(D)**. DAP0, DAP8, DAP10, DAP12, DAP20, DAP30, and DAP40 represent 0, 8, 10, 12, 20, 30, and 40 d after pollination, respectively.
