# Supplementary material for: Transcriptomic Variations and Network Hubs Controlling Seed Size and Weight During Maize Seed Development
Source: Front Plant Sci. 2022 Feb 14;13:828923. doi: 10.3389/fpls.2022.828923 (PMC8882617; doi:10.3389/fpls.2022.828923)
Supplement: Supplementary file 2 [file Table_1.docx]

**Supplementary Table 1** **Analysis of variances of seed size and weight associated traits for eight genotypes at seven developmental stages**

| Traits^†^ | Sources^‡^ | DF^§^ | SS^¶^ | MS* | F value | *P* value |
| --- | --- | --- | --- | --- | --- | --- |
| FKW | Genotype | 7 | 419 | 60 | 149.657 | <2E-16 |
|  | DAP | 5 | 27956 | 5591 | 13967.1 | <2E-16 |
|  | Replication | 2 | 1 | 0 | 0.887 | 0.415 |
|  | Genotype: DAP | 35 | 172 | 5 | 12.288 | <2E-16 |
|  | Residuals | 94 | 38 | 0 |  |  |
| DKW | Genotype | 7 | 57 | 8.2 | 53.088 | < 2E-16 |
|  | DAP | 5 | 12668 | 2533.6 | 16397.26 | < 2E-16 |
|  | Replication | 2 | 2 | 0.8 | 4.863 | 0.0098 |
|  | Genotype: DAP | 35 | 32 | 0.9 | 5.908 | 3.16E-12 |
|  | Residuals | 94 | 15 | 0.2 |  |  |
| TKL | Genotype | 7 | 59.7 | 8.53 | 423.881 | <2E-16 |
|  | DAP | 5 | 560.3 | 112.07 | 5568.515 | <2E-16 |
|  | Replication | 2 | 0.1 | 0.04 | 1.951 | 0.15 |
|  | Genotype: DAP | 35 | 22.4 | 0.64 | 31.791 | <2E-16 |
|  | Residuals | 94 | 1.9 | 0.02 |  |  |
| TKW | Genotype | 7 | 24.5 | 3.5 | 172.301 | <2E-16 |
|  | DAP | 5 | 440.6 | 88.11 | 4343.801 | <2E-16 |
|  | Replication | 2 | 0.1 | 0.03 | 1.502 | 0.23 |
|  | Genotype: DAP | 35 | 21.9 | 0.63 | 30.888 | <2E-16 |
|  | Residuals | 94 | 1.9 | 0.02 |  |  |

^†^ FKW, 100-fresh kernel weight; DKW, 100-dry kernel weight; TKL, ten-kernel length; TKW, ten-kernel width. ^‡^ DAP, d after pollination; ^§^ DF, degree of freedom; ^¶^ SS, sum of square; * MS, mean of square.

| **Supplementary Table 2 List of primers used in this study** | |
| --- | --- |
| primer | sequence ^†^ (5'->3') |
| ARF12-VIGS-F | GT*CCATGGG*CGTTTGACAAATTTGTAATGT |
| ARF12-VIGS-R | GT*CCTAGG*CAACAAATGGTACCAACAGG |
| IAA8-VIGS-F | AT*CCATGG*CGTGTGTACAGTTGTCGCCT |
| IAA8-VIGS-R | TA*CCTAGG*GAGATGGCAAAGCAACCGTG |
| ARF12-qRT-F | TTCCGAGGAGGATGTTTGGC |
| ARF12-qRT-R | ACATGCCTACCGTCAGGTTG |
| ARF23-qRT-F | TCCTGCAAGGAACGCTGAAT |
| ARF23-qRT-R | GAACCGTGCCCAAACAAGTC |
| ARF24-qRT-F | TCAGAACACCACTCGGAAGC |
| ARF24-qRT-R | ACTCAAGCTTGCATCGACCA |
| IAA8-qRT-F | ATGTTCGTGGAAACCTGCCA |
| IAA8-qRT-R | CCCTTTCCTTCAAGACGCCA |
| ZmUbi-qRT-F | GGAAAAACCATAACCCTGGA |
| ZmUbi-qRT-R | ATATGGAGAGAGGGCACCAG |
| ^†^ The restriction sites are shown in italic type. | |
